# Efficacy of platelet-rich plasma in the treatment of erectile dysfunction: A meta-analysis of controlled and single-arm trials

**DOI:** 10.1371/journal.pone.0313074

**Published:** 2024-11-14

**Authors:** Shaokang Du, Shiwei Sun, Fuyu Guo, Hongyao Liu

**Affiliations:** 1 Department of Urology, Third Hospital of Shanxi Medical University, Shanxi Bethune Hospital, Shanxi Academy of Medical Sciences, Tongji Shanxi Hospital, Taiyuan, China; 2 Department of Urology, Peking Union Medical College Hospital, Chinese Academy of Medical Sciences and Peking Union Medical College, Beijing, China; Shiraz University of Medical Sciences, ISLAMIC REPUBLIC OF IRAN

## Abstract

**Background:**

Erectile dysfunction (ED) is a prevalent condition in urology, and studies on the effectiveness of platelet-rich plasma (PRP) for this condition have been conducted; however, the evidence remains inconclusive. This meta-analysis aimed to evaluate the effectiveness of PRP in treating ED.

**Methods:**

On May 17, 2024, a literature search was performed and evaluated using the Cochrane method. The primary outcome measured was the International Index of Erectile Function (IIEF) score, while the secondary outcomes included Minimal Clinically Important Difference (MCID) and peak systolic velocity (PSV).

**Results:**

A total of 12 controlled trials involving 991 patients and 11 single-arm trials with 377 patients were analyzed. The findings revealed that compared to the control group, the PRP group demonstrated better outcomes in terms of the IIEF score and MCID (SMD = 0.59 (95% CI: [0.34, 0.84]; RR = 1.94 (95% CI: [1.33, 2.83]), In the single-arm trials, a significant improvement in IIEF scores was observed following PRP treatment (SMD = -0.99 95% CI: [-1.53, 0.46]).

**Conclusion:**

PRP appears effective in treating erectile dysfunction, but further high-quality, large-sample trials with longer follow-up are needed to fully understand its effects.

## Introduction

Erectile dysfunction, a prevalent condition in urology, is characterized by the difficulty of achieving or maintaining erection hardness during sexual activity. This condition significantly affects one’s ability to engage in a satisfactory sex life. Additionally, various health issues such as obesity, diabetes, and depression have been identified as contributing factors to the development of erectile dysfunction (ED) [1]. While various treatments are available for the disease, such as oral medications, extracorporeal shockwave therapy (ESWT) [2], and the placement of penile prostheses [3], the majority of these options primarily focus on enhancing hemodynamics. However, it is important to note that there remains a significant gap in treatments that specifically target the reversal of the pathophysiology underlying ED [4].

Platelet-rich plasma (PRP) is a type of plasma derived from blood centrifugation, with a higher platelet concentration than autologous blood. PRP contains significant amounts of growth factors such as platelet-derived growth factor (PDGF), transforming growth factor-β (TGF-β), and vascular endothelial growth factor (VEGF) [5]. PDGF promotes the repair and remodeling of penile vasculature by stimulating the proliferation of vascular smooth muscle cells and fibroblasts, thereby enhancing blood flow and vascular health, which positively affects erectile function [6]. TGF-β regulates inflammatory responses and promotes collagen synthesis, aiding in the repair of damaged tissues, reducing fibrosis, and improving the elasticity and function of penile tissue [7]. VEGF enhances endothelial cell proliferation and angiogenesis, increasing penile blood flow and directly improving erectile capacity [8]. These growth factors work synergistically to promote tissue regeneration and angiogenesis, collectively improving symptoms of ED.

Recent studies have suggested that PRP could be a promising treatment for ED. However, the experimental outcomes have been inconsistent. Therefore, this study aimed to perform a meta-analysis of existing clinical trials to assess the effectiveness of PRP in the treatment of ED.

## Method

### Literature screening and inclusion criteria

During the study selection process, two investigators were responsible for identifying eligible studies by adhering to specific inclusion and exclusion criteria. The inclusion criteria for this study are randomized controlled trials and single-arm trials involving ED patients treated with PRP, where indicators such as the International Index of Erectile Function (IIEF) score are evaluated before and after treatment, with both types of trials undergoing independent meta-analyses. Any disagreements between the two investigators were resolved through discussions or with the assistance of a third party. Exclusion criteria comprised duplicate publications, non-clinical trials, case reports, systematic reviews, non-English literature, and studies with incomplete or unavailable data. Research with missing data on the outcome measure were excluded. Notably, this study was registered in PROSPERO: CRD42024547695.

### Literature search strategy

In May 2024, we conducted a systematic search of several databases including PubMed, Web of Science, Medline, Embase, Cochrane Library, and ClinicalTrials.gov, using MeSH terminology and a specific search formula. The detailed search string is presented in the Supplement (S1 File).

### Ethics statement

Databases such as PubMed and MEDLINE are public databases. The patients involved in the database have obtained ethical approval. Users can download relevant data for free for research and publish relevant articles. Our study is based on open source data, so there are no ethical issues and other conflicts of interest.

### Analysis of bias in the included studies

The Cochrane risk-of-bias assessment tool [9] was applied, consisting of six components: random sequence generation, allocation concealment, blinding of participants and personnel, incomplete outcome data, selective reporting, and other biases. Disagreements among researchers were resolved through discussion to ensure consensus, and a risk of bias table was then created using RevMan 5.4 (Review Manager, Version 5.4, The Cochrane Collaboration, 2020).

### Data extraction and analysis

Data extraction involved collecting basic information on the articles and relevant outcome measures. Mean and standard deviation were utilized for continuous variables, while median, quartile, and range data were adjusted according to Wan et al.’s formulas [10, 11]. The data synthesis process utilized the RevMan 5.4 software program, alongside forest plots and funnel plots.

The primary outcome measure in this study was the IIEF score. Secondary indicators, including Minimal Clinically Important Difference (MCID), Peak Systolic Velocity (PSV)., End Diastolic Velocity (EDV), Visual Analog Scale (VAS), and 95% confidence intervals (CI), were considered. For the analysis of continuous variables such as the IIEF score, the researchers selected the 95% confidence intervals and utilized the inverse variance method (IV). In contrast, for dichotomous variables, relative risk (RR) was employed.

### Heterogeneity assessment, subgroup analysis and publication bias

The Cochran’s chi-square test was utilized to detect heterogeneity among the included studies, with heterogeneity being assessed based on a p-value of <0.05. Study differences were quantified using the I2 statistic, with a random-effects model applied for heterogeneity exceeding 50%, and a fixed-effects model for heterogeneity below 50%. Subgroup analyses were conducted based on whether PRP was combined with the other treatment modalities. Funnel plots were generated using RevMan5, and the Begg and Egger methods were employed to ascertain publication bias. Additionally, to evaluate the stability of results with heterogeneity greater than 50% (I^2^>50%) and to identify the source of heterogeneity, STATA 18.0, was utilized.

## Result

**Fig 1:**
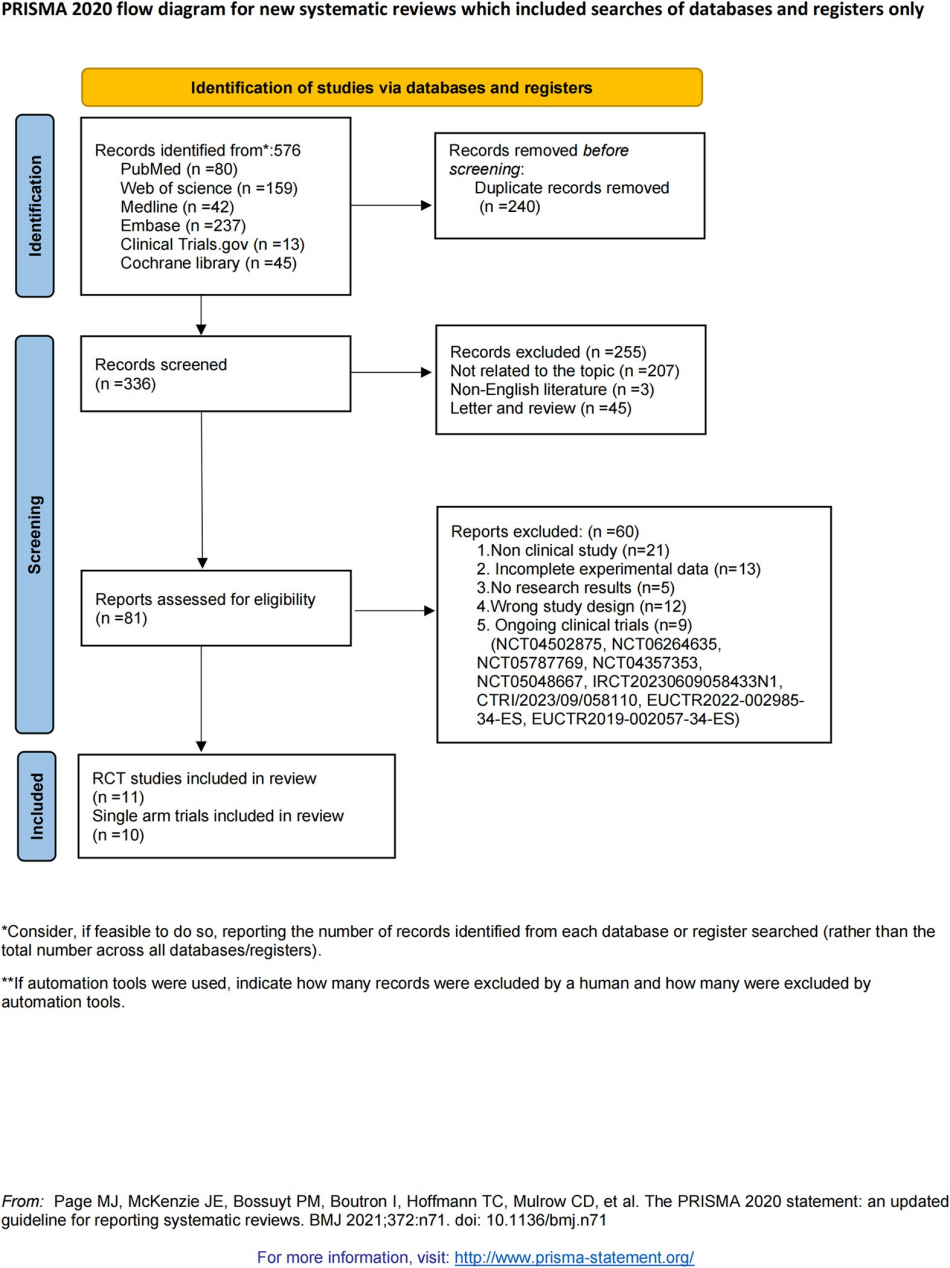
Flow chart of study selection.

A total of 576 articles were retrieved. After eliminating 240 duplicates, 255 non-conforming articles were excluded based on the abstracts (S1 Table). Subsequently, 60 articles were excluded for various reasons after reviewing the full text of the remaining literature, ultimately including 11 randomized controlled experiments and 10 single-arm experiments in the analysis (Fig 1), both of which will undergo independent meta-analyses (S2 Table).

### Literature characteristics

We included a total of 21 articles in our analysis [12–32], comprising 11 controlled trials involving 991 patients [12–22]. These studies were geographically diverse, with three conducted in Italy [12, 13], two in the USA [15, 16], two in Turkey [17, 18], and one each in Egypt [19], India [20], Greece [21], and Russia [22]. Five studies utilized PRP as the sole treatment [15, 16, 19–21], while the remaining studies combined PRP with ESWT. Among these, there were 10 prospective studies and one retrospective [18] study. In addition to the controlled trials, 10 single-arm trials were included in the analysis, totaling 377 patients [23–32]. All single-arm trials were prospective studies, with two conducted in Russia [23, 24], two in Italy [25, 26], two in France [27, 28], and one each in Egypt [29], Turkey [30], China [31], and Morocco [32]. Among the single-arm trials, two studies combined PRP with another treatment modality [23, 24], while the rest focused solely on PRP therapy.

### Risk of study bias analysis

**Fig 2:**
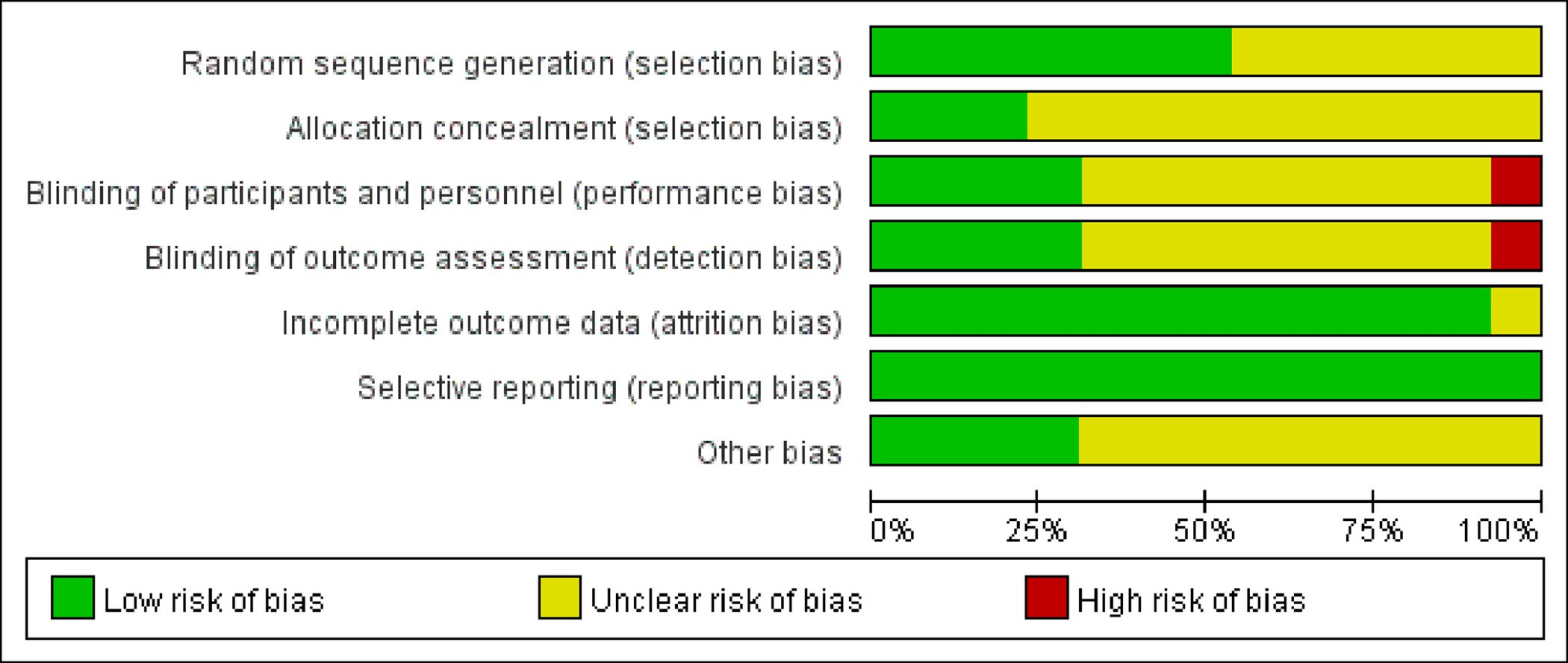
Risk of bias map for randomized controlled trials.

All included studies were found to have a risk of bias using the Cochrane risk-of-bias assessment tool. The main sources of bias identified were missing data and result measurement issues (S3 Table). In the combination therapy group, the main source of bias was the inability to blind patients (Fig 2).

### Analysis of results

#### IIEF score

After analyzing data from 10 studies involving 932 patients, with 463 (49.7%) in the PRP group and 469 (50.3%) in the control group (placebo and non-PRP), a significant difference was observed between the PRP and control groups (SMD = 0.59, 95% CI: [0.34, 0.84]).

**Fig 3:**
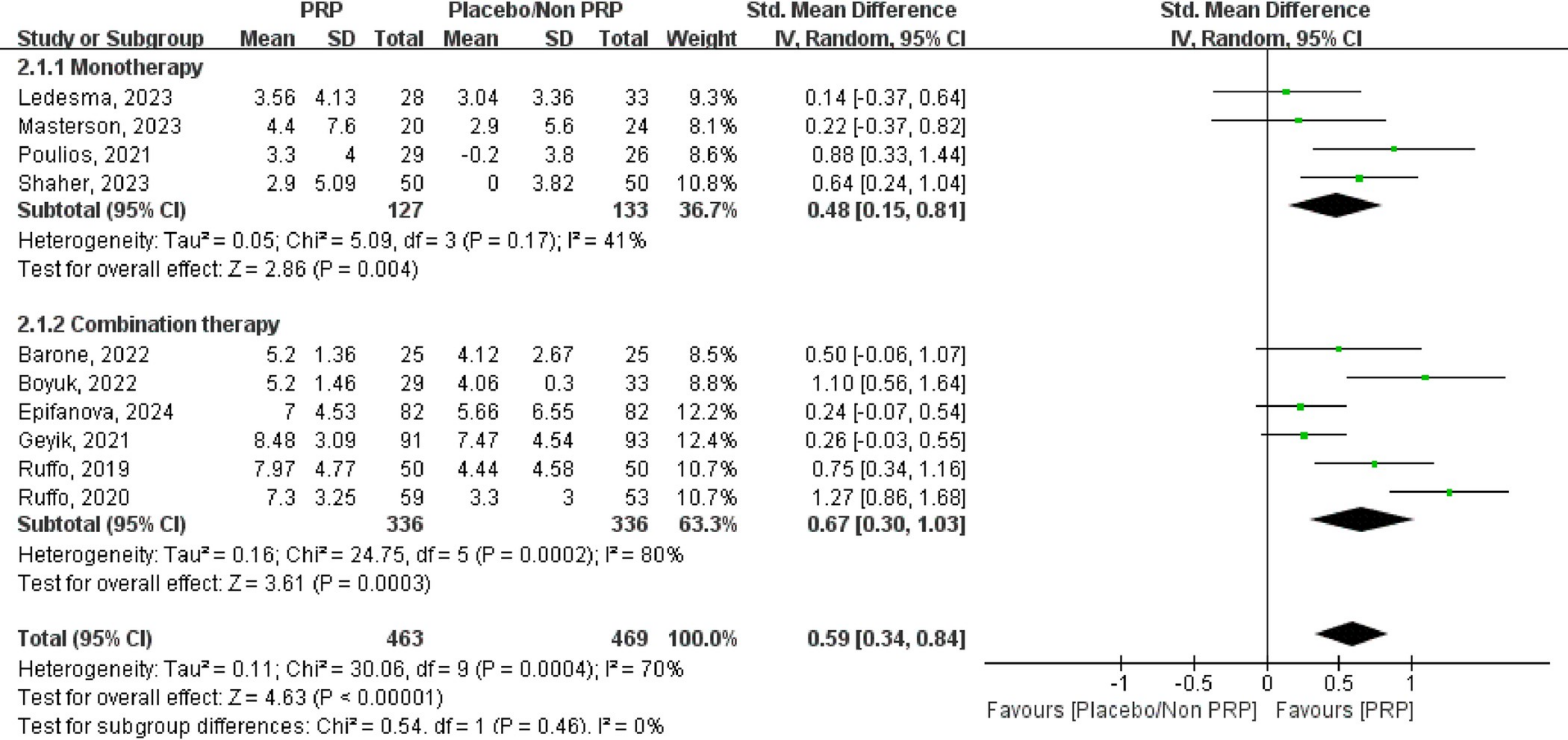
The forest plot illustrates platelet-rich plasma (PRP) vs placebo/Non platelet-rich plasma (Non PRP) in change in the International Index of Erectile Function (IIEF) score.

**Fig 4:**
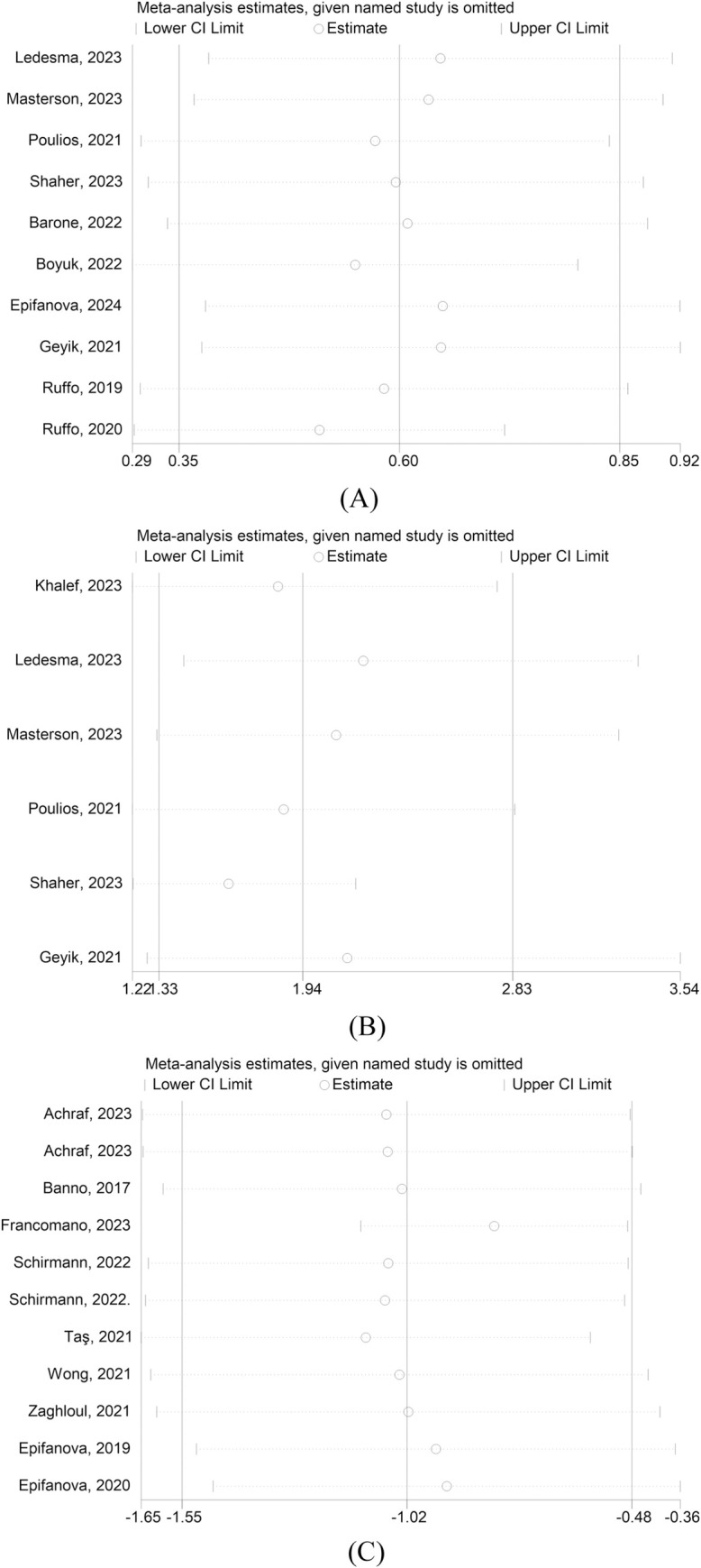
Sensitivity analysis results. 4-A: The sensitivity analvsis of llEF score. 4-B: The sensitivity analysis of MClD 4-C: The sensitivity analysis of llEF score in single-arm group.

In the monotherapy subgroup, comprising 4 studies with 260 patients (127 in the PRP group and 133 in the control group), a significant difference was also observed between the PRP and control groups (SMD = 0.48, 95% CI: [0.15, 0.81]). Similarly, in the combination therapy subgroup, which included 6 studies with 672 patients, evenly split between the PRP group and the control group (336 each, 50.0%), a significant difference was noted between the two groups (SMD = 0.67, 95% CI: [0.30, 1.03]) (Fig 3). Despite the high heterogeneity in the studies, the sensitivity analysis confirmed that the results remained stable and reliable (Fig 4A).

The correlation between effect size and publication year in PRP treatment studies for ED is shown in the Supplement (S1 Fig). The vertical axis represents effect size, with each circle depicting a study. Larger circles indicate smaller variance, higher precision, and greater weight in the meta-analysis. The horizontal axis marks the publication year.

In the single-arm trial, a total of 11 studies involving 377 patients revealed a statistically significant difference in IIEF scores after PRP treatment compared to scores before treatment (SMD = -0.99 95% CI: [-1.53, -0.46]) (S2 Fig). High study heterogeneity was observed, but the results remained stable after the sensitivity analysis (Fig 4C).

#### MCID

In the analysis of 6 studies involving 503 patients, 260 (51.7%) were in the PRP group and 243 (48.3%) in the control group (placebo and non-PRP). The PRP group demonstrated a significantly higher MCID compared to the control group (RR = 1.94, 95% CI: [1.33, 2.83]), further confirming the advantage of PRP (RR = 2.13, 95% CI: [1.28, 3.54]; RR = 1.50, 95% CI: [1.17, 1.92]) (S3 Fig). Notably, the results exhibited high heterogeneity, primarily attributable to the study conducted by Shaher, 2023. Upon excluding this study, the increased MCID in the PRP group remained statistically significant (RR = 1.62, 95% CI: [1.22, 2.16]) (S4 Fig). The sensitivity analyses indicated the stability of these results (Fig 4B).

#### PSV and EDV

In 6 studies involving 581 patients, with 289 (49.7%) in the PRP group and 292 (50.3%) in the control group, PSV was assessed, showing a significantly higher PSV in the PRP group compared to the control group (SMD = 1.12, 95% CI: [0.45, 1.79]).

In the monotherapy subgroup, consisting of 3 studies with 205 patients (98 in the PRP group and 107 in the control group), the advantage of PSV in the PRP group was not sustained (SMD = 1.14, 95% CI: [-0.40, 2.67]). However, in the combination therapy subgroup, which included 3 studies with 376 patients (191 in the PRP group and 185 in the control group), a significant difference was observed between the PRP and control groups (SMD = 1.09, 95% CI: [0.42, 1.77]) (S5 Fig).

In the single-arm trial, the pooled effect sizes indicated a significant difference in PSV before and after PRP treatment (SMD = -0.88, 95% CI: [-1.73, -0.02]), but not in the monotherapy subgroup (SMD = -0.86 95% CI: [-2.35, 0.62]) (S6 Fig).

Two studies with a total of 161 patients in EDV found no significant difference between PRP and control groups (SMD = -1.28, 95% CI: [-4.22, 1.67]) (S7 Fig).

#### VAS

In the analysis of 3 studies involving 199 patients (99 in the PRP group and 100 in the control group), VAS scores were examined, showing no statistically significant difference between the PRP and control groups (SMD = -0.23, 95% CI: [-0.72, 0.25]) (S8 Fig).

#### Complication

Only 4 complications were identified in the trials: Masterson et al. reported 1 plaque in the PRP group and 1 hematoma in the placebo group, while Boyuk reported 2 ecchymoses in the PRP treatment group.

## Heterogeneity analysis and publication bias

The pooled effect size analysis of the primary outcome IIEF score indicated high heterogeneity, potentially attributable to varying combinations in the combination drug group. Despite this, the statistical significance persisted even after sensitivity analysis, suggesting robust and stable results. In a single-arm experimental analysis of IIEF scores, Francomano, 2023 [27] emerged as the primary source of heterogeneity within the monotherapy subgroup. Upon its removal, the heterogeneity notably decreased, indicating a significant impact on the overall results, which demonstrated that the IIEF scores following PRP treatment remained statistically different from baseline scores (SMD = -0.78, 95% CI: [-1.09, -0.47]) (S9 Fig). The study by Shaher, 2023 [22] was identified as the main source of heterogeneity in the MCID results. Following the exclusion of this study, the heterogeneity significantly decreased, yet the pooled effect size remained statistically significant.

A bias assessment was conducted for the primary outcome measure rows, including the funnel plots (S10 Fig). Egger’s test: P>|t| = 0.301 and Begg’s test: Pr>|z| = 0.474, indicating no publication bias present.

## Discussion

PRP is gaining popularity in regenerative medicine, particularly in orthopedics and other medical specialties, owing to its easy procurement, safety profile, and minimal adverse effects [33, 34]. However, there is a scarcity of clinical evidence supporting the utilization of PRP in urology.

In 2012, Wu et al. [35] demonstrated that the injection of PRP in rats with bilateral cavernous nerve crush injury resulted in the promotion of nerve myelin axon regeneration and the restoration of ED. From 2012 to 2017, Matz [36] conducted the first clinical trial of PRP in the treatment of ED. They reported an average improvement in IIEF scores of 4.14 points among patients treated with PRP. Moreover, 80 percent of patients expressed a willingness to continue treatment, and no complications were observed during follow-up. Subsequently, in 2021, Taş [30] evaluated the therapeutic effect of PRP in ED patients with metabolic syndrome, observing a 5-point increase in patients’ IIEF scores post-injection. Poulios et al.’s placebo-controlled experiment in 2021 indicated superior outcomes in the PRP group compared to the placebo group, emphasizing the potential benefits of PRP therapy. These studies collectively highlight the potential efficacy of PRP in treating ED. However, despite these promising findings, there are limitations in the current body of research on PRP for ED treatment. In contrast, a study by Ragheb et al. [37] in 2024 revealed conflicting results, wherein patients treated with PRP showed a lower change in IIEF scores than the placebo group. These contradictory findings underscore the need for further investigations to establish the definitive efficacy of PRP in ED treatment. Specifically, larger multicenter trials are necessary to address the limited sample sizes of existing studies and verify the therapeutic potential of PRP. Moreover, the high cost associated with PRP treatment poses a significant economic burden on patients and warrants consideration in the evaluation of its overall utility for managing ED [38]. More robust experimental data are crucial for resolving the current inconsistencies surrounding the use of PRP in ED therapy.

This study presents several sources of bias, including missing data and result measurement issues, which can be addressed by employing multiple imputation to estimate missing values and implementing rigorous follow-up to minimize data loss; however, the risk of bias remains if the missing data are not at random [39]. Standardizing measurement protocols and utilizing validated instruments are recommended to reduce measurement bias, although variations in execution may still introduce discrepancies [40, 41]. While placebo controls or double-dummy techniques can mitigate bias in patient blinding, achieving complete blinding may still be impossible if noticeable differences between treatments exist, potentially leading to residual bias [42, 43].

In this study, we observed that patients treated with PRP showed higher IIEF scores than those who did not receive PRP (SMD = 0.59, 95% CI: [0.34, 0.84]), both in monotherapy and combination therapy modalities (SMD = 0.48, 95% CI: [0.15, 0.81] SMD = 0.67, 95% CI: [0.30, 1.03]). The IIEF score, serving as the primary indicator in our investigation, is crucial for evaluating erectile function, with a heightened score suggesting an improvement in patient symptoms [44]. Although the study displayed a high level of heterogeneity, it maintained an acceptable level of stability, with the monotherapy group not exhibiting significant heterogeneity. Masterson et al. noted in their study that patients in the placebo group were permitted to use phosphodiesterase-5 (PDE5) inhibitors during the trial and received lower PRP injections, potentially explaining the lack of statistically significant outcomes between the experimental and control cohorts.

Our study found that the combination of PRP+ESWT and PRP therapy following ESWT led to significantly higher results compared to ESWT alone, a conventional treatment approach known for its ability to stimulate cell proliferation, tissue regeneration, and angiogenesis, similar to PRP [45, 46]. The data revealed that PRP administration during ESWT further enhanced patients’ IIEF scores, resulting in a more effective therapeutic outcome (SMD = 0.67, 95% CI: [0.30, 1.03]). Moreover, in single-arm trial, we observed a notable enhancement in patients’ IIEF scores post PRP treatment in comparison to pretreatment levels, consistent with results from the control group. Similarly, according to a study by Sajjad [47], both PRP and ESWT treatments resulted in increased IIEF scores without a statistically significant difference, indicating an improvement in patients’ psychological well-being, which is consistent with our study findings.

The declining trend in the correlation graph suggests that newer studies report smaller effects, possibly due to more rigorous designs, larger sample sizes, or stricter methodologies in recent research.

In this study, MCID and PSV showed statistically significant improvements following PRP treatment. MCID suggests that patients perceive noticeable benefits in erectile function after PRP treatment, while the improvement in PSV indicates enhanced penile blood flow during erection. However, no significant differences were observed in EDV and VAS. The lack of change in EDV may suggest that PRP’s vascular benefits are more pronounced during erection rather than at rest. Similarly, the unchanged VAS scores imply that the PRP group experienced no significant difference in treatment-related pain compared to the placebo group. Nevertheless‌, these results may stem from limitations in the included literature, necessitating further comprehensive research [16, 19, 21].

Out of the included literature, only 2 studies reported 4 minor complications related to intracavernosal PRP, indicating its safety.

Recent studies have demonstrated promising results in enhancing erectile function in patients with penile deformities. We did not analyze changes in penile angle pre- and post-PRP treatment due to the limited number of relevant studies. Zugail’s [48] study found that following PRP treatment, the angle of penile curvature decreased from 45° (40°-75°) to 30° (20°-40°). Moreover, combining PRP with hyaluronic acid, as demonstrated by Virag [49], resulted in a significant 36.9% reduction in the angle of penile curvature, with 82.7% of patients reporting improvements in erection quality. These findings indicate the potential therapeutic benefits of PRP for erectile function in patients with penile deformities. However, despite these positive results, further research is necessary to establish the efficacy of PRP treatment in this context, highlighting the need for additional data in future studies.

PRP, as a new drug, provides a long-term, stable treatment modality compared with pre-coital medication, long-term injection can enhance patient confidence and may be suitable for those patients with psychological ED or those who are unwilling to take oral drugs, despite its slow onset of action compared with first-line treatment like PED5i [50]. Different studies have shown that variations in the preparation or activation methods of PRP can lead to different effects [51]. Moreover, the growth factor content in PRP varies among different populations, including ED patients [52, 53]. As the preparation and injection of PRP are tailored to individual patients, differences in growth factor content can result in varying therapeutic outcomes. Therefore, selecting the most suitable preparation or activation method based on the patient’s specific condition is crucial for conducting personalized treatment approaches.

In recent years, a variety of treatments for ED have been developed, such as ESWT, stem cells, and PRP as investigated in this study. Studies have demonstrated that these treatments offer various advantages when administered independently or in conjunction with first-line therapies [54, 55]. However, it is premature to implement widespread clinical treatment based solely on these findings. Further research involving high-quality randomized controlled trials is essential to validate and establish the effectiveness of these emerging treatments.

In our meta-analysis, PRP combined with ESWT was analyzed as a subgroup, aligned with the methodology of previous studies that focused on primary outcomes [56]. Although certain sources were presented as conference abstracts, they were integrated into the analysis to provide additional evidence. The results of our investigation revealed a mixture of heterogeneous yet consistent findings. Notably, some studies in the combination group exhibited bias due to the inability to blind patients. Furthermore, a majority of the trials featured short-term follow-up periods, which hindered the evaluation of the long-term effects of PRP, either alone or in conjunction with ED. Regrettably, due to the limited number of studies, subgroup analyses based on the type of ED were not feasible.

## Conclusion

This study indicated that PRP, either alone or in combination with ESWT, is a safe and effective treatment option for patients with ED. However, due to significant heterogeneity in the current studies, further research involving longer follow-up and high-quality, large-sample clinical trials are necessary to better understand the effects of PRP.

## Supporting information

S1 Checklist PRISMA 2020 checklist. (DOCX)

S1 File Search string. (DOCX)

S1 Fig Correlation graph between effect size and publication year in PRP treatment studies for ED. (TIF)

S2 Fig The forest plot shows the changes in platelet rich plasma (PRP) in the International Erectile Function Index (IIEF) score during a single-arm trial. (TIF)

S3 Fig The forest plot illustrates platelet-rich plasma (PRP) vs placebo/Non platelet-rich plasma (Non PRP) in change in achieving minimal clinically important difference (MCID). (TIF)

S4 Fig The forest plot illustrates platelet-rich plasma (PRP) vs placebo/Non platelet-rich plasma (Non PRP) in change in achieving minimal clinically important difference (MCID) without Shaher, 2023. (TIF)

S5 Fig The forest plot illustrates platelet-rich plasma (PRP) vs placebo/Non platelet-rich plasma (Non PRP) in peak systolic velocity (PSV). (TIF)

S6 Fig The forest plot shows the changes in platelet rich plasma (PRP) in peak systolic velocity (PSV) during a single-arm trial. (TIF)

S7 Fig The forest plot illustrates platelet-rich plasma (PRP) vs placebo in end-diastolic velocity (EDV). (TIF)

S8 Fig The forest plot illustrates platelet-rich plasma (PRP) vs placebo in visual analog scale (VAS) score. (TIF)

S9 Fig The forest plot shows the changes in platelet rich plasma (PRP) in the International Erectile Function Index (IIEF) score during a single-arm trial without Francomano, 2023. (TIF)

S10 Fig IIEF score publication bias funnel plot. (TIF)

S1 Table All studies identified in the literature search with reasons for exclusion. (DOCX)

S2 Table Data extracted from primary research sources for systematic review and/or meta-analysis. (DOCX)

S3 Table Completed risk of bias and quality/certainty assessments for each study or outcome. (DOCX)
