# Supplementary material for: Efficacy of platelet-rich plasma in the treatment of erectile dysfunction: A meta-analysis of controlled and single-arm trials
Source: PLoS One. 2024 Nov 14;19(11):e0313074. doi: 10.1371/journal.pone.0313074 (PMC11563399; doi:10.1371/journal.pone.0313074)
Supplement: S1 File — (DOCX) [file pone.0313074.s002.docx]

**Search string**

("platelet rich plasma"[MeSH Terms] OR ("platelet rich"[All Fields] AND "plasma"[All Fields]) OR "platelet rich plasma"[All Fields] OR ("plasma"[All Fields] AND "platelet"[All Fields] AND "rich"[All Fields]) OR "plasma platelet rich"[All Fields] OR ("platelet rich plasma"[MeSH Terms] OR ("platelet rich"[All Fields] AND "plasma"[All Fields]) OR "platelet rich plasma"[All Fields] OR ("platelet"[All Fields] AND "rich"[All Fields] AND "plasma"[All Fields]) OR "platelet rich plasma"[All Fields])) AND ("erectile dysfunction"[MeSH Terms] OR ("erectile"[All Fields] AND "dysfunction"[All Fields]) OR "erectile dysfunction"[All Fields] OR ("dysfunction"[All Fields] AND "erectile"[All Fields]) OR "dysfunction erectile"[All Fields] OR ("erectile dysfunction"[MeSH Terms] OR ("erectile"[All Fields] AND "dysfunction"[All Fields]) OR "erectile dysfunction"[All Fields] OR ("male"[All Fields] AND "impotence"[All Fields]) OR "male impotence"[All Fields]) OR ("erectile dysfunction"[MeSH Terms] OR ("erectile"[All Fields] AND "dysfunction"[All Fields]) OR "erectile dysfunction"[All Fields] OR ("impotence"[All Fields] AND "male"[All Fields]) OR "impotence male"[All Fields]) OR ("erectile dysfunction"[MeSH Terms] OR ("erectile"[All Fields] AND "dysfunction"[All Fields]) OR "erectile dysfunction"[All Fields] OR ("male"[All Fields] AND "sexual"[All Fields] AND "impotence"[All Fields]) OR "male sexual impotence"[All Fields]) OR ("erectile dysfunction"[MeSH Terms] OR ("erectile"[All Fields] AND "dysfunction"[All Fields]) OR "erectile dysfunction"[All Fields] OR ("impotence"[All Fields] AND "male"[All Fields] AND "sexual"[All Fields])) OR ("erectile dysfunction"[MeSH Terms] OR ("erectile"[All Fields] AND "dysfunction"[All Fields]) OR "erectile dysfunction"[All Fields] OR ("sexual"[All Fields] AND "impotence"[All Fields] AND "male"[All Fields])) OR ("erectile dysfunction"[MeSH Terms] OR ("erectile"[All Fields] AND "dysfunction"[All Fields]) OR "erectile dysfunction"[All Fields] OR "impotence"[All Fields] OR "impotent"[All Fields] OR "impotency"[All Fields]))
