# Supplementary figures and images for: Efficacy of platelet-rich plasma in the treatment of erectile dysfunction: A meta-analysis of controlled and single-arm trials
Source: PLoS One. 2024 Nov 14;19(11):e0313074. doi: 10.1371/journal.pone.0313074 (PMC11563399; doi:10.1371/journal.pone.0313074)

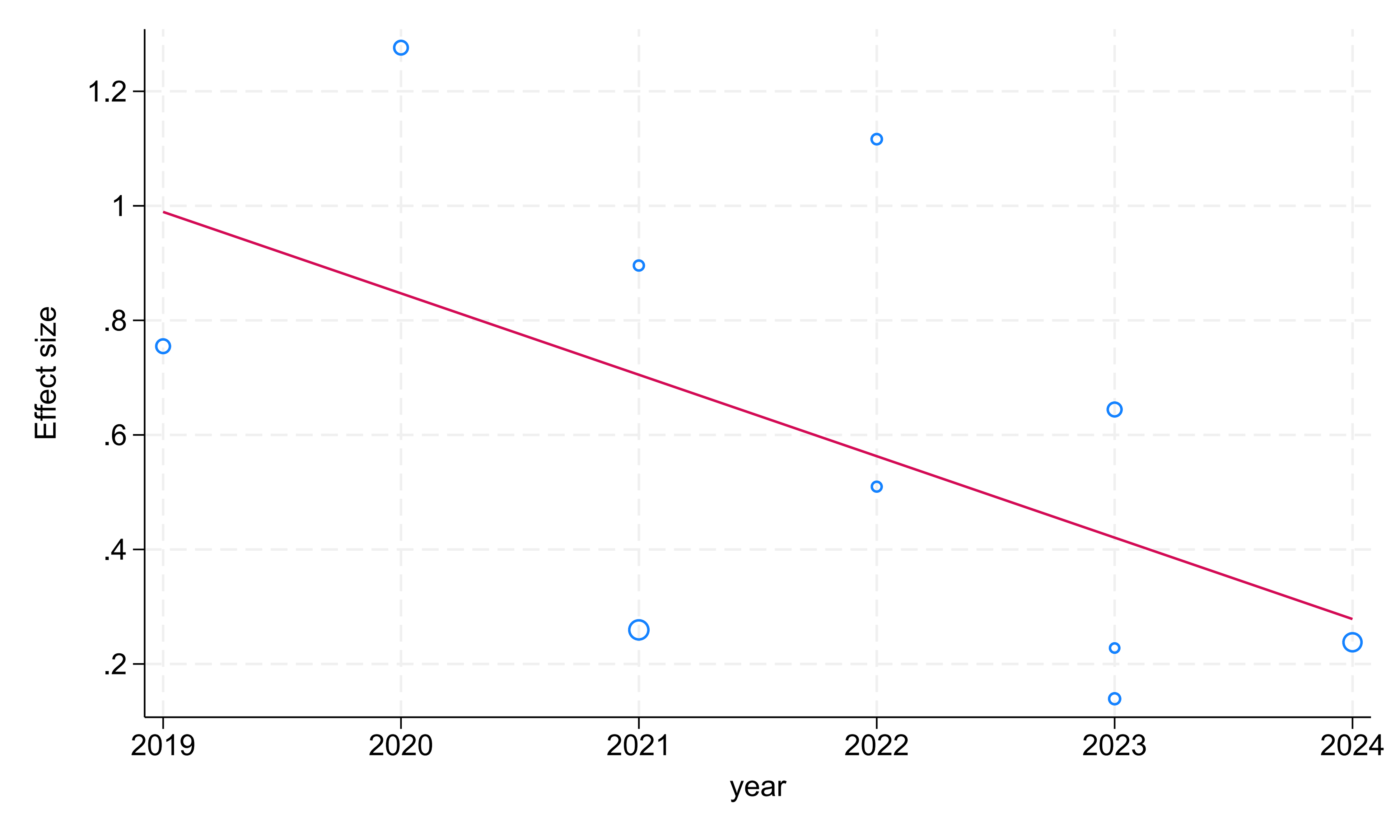

Supplement: S1 Fig — (TIF) [file pone.0313074.s003.tif]

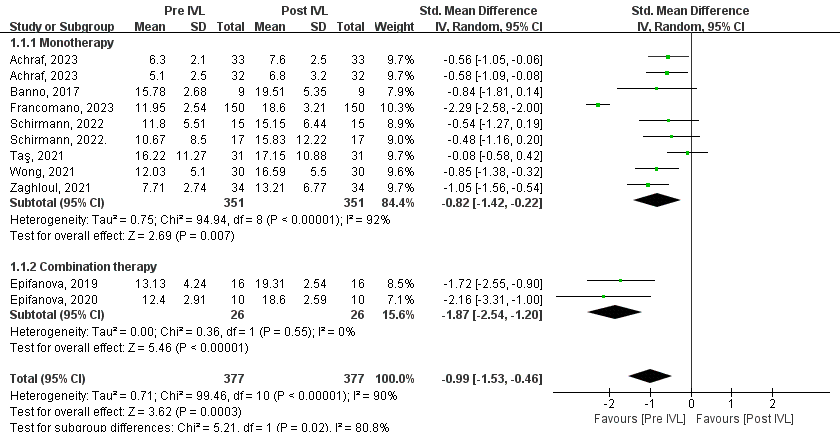

Supplement: S2 Fig — (TIF) [file pone.0313074.s004.tif]

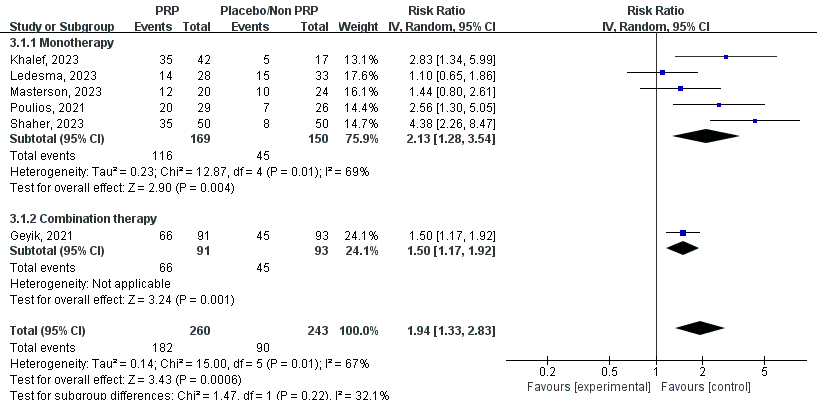

Supplement: S3 Fig — (TIF) [file pone.0313074.s005.tif]

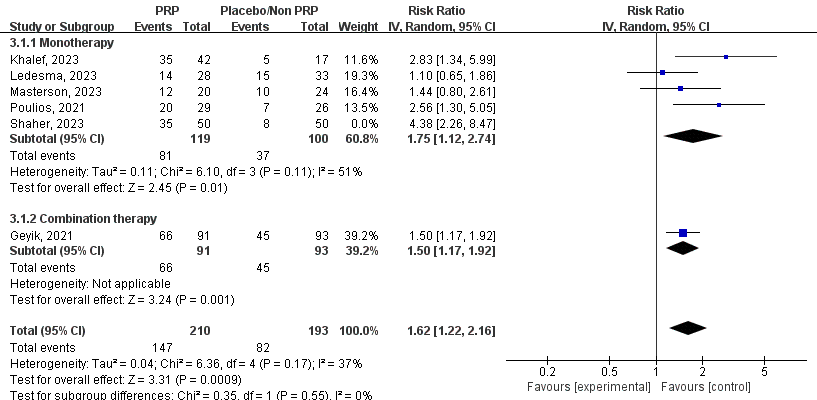

Supplement: S4 Fig — (TIF) [file pone.0313074.s006.tif]

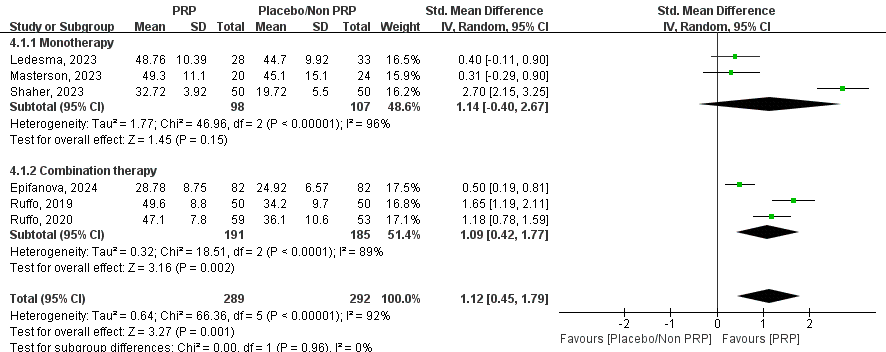

Supplement: S5 Fig — (TIF) [file pone.0313074.s007.tif]

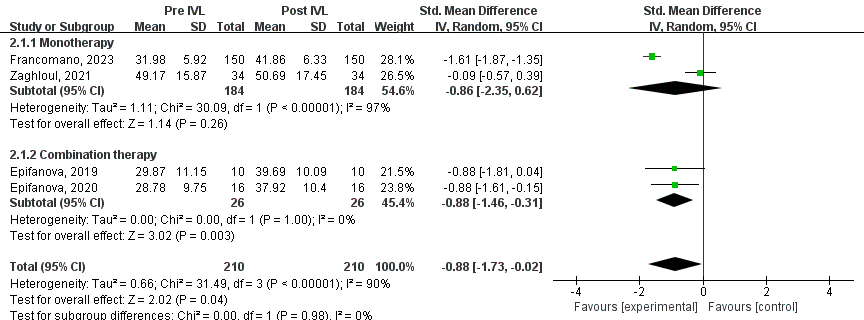

Supplement: S6 Fig — (TIF) [file pone.0313074.s008.tif]

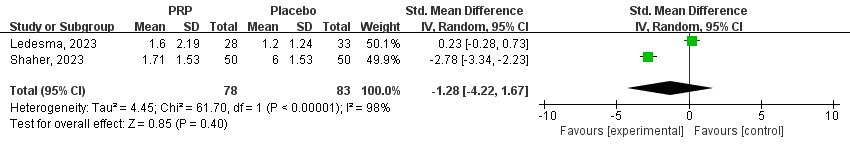

Supplement: S7 Fig — (TIF) [file pone.0313074.s009.tif]

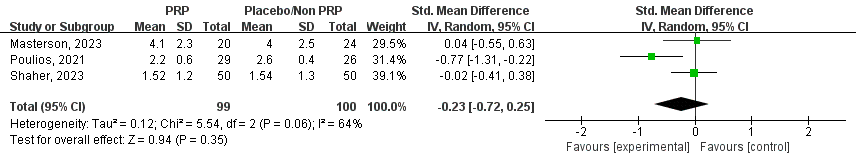

Supplement: S8 Fig — (TIF) [file pone.0313074.s010.tif]

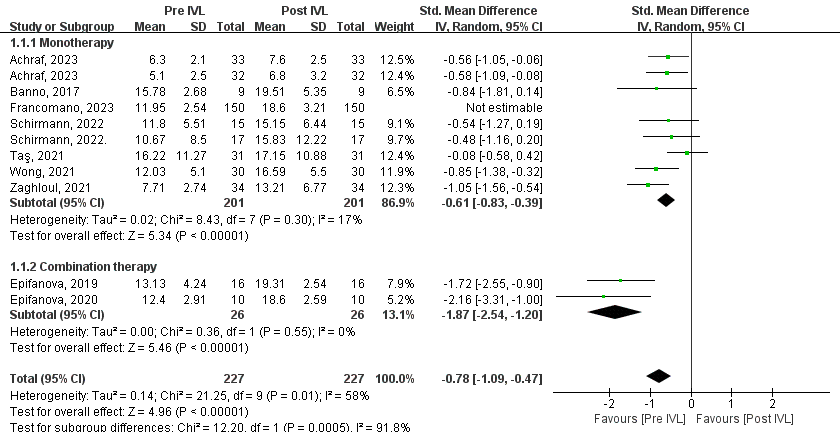

Supplement: S9 Fig — (TIF) [file pone.0313074.s011.tif]

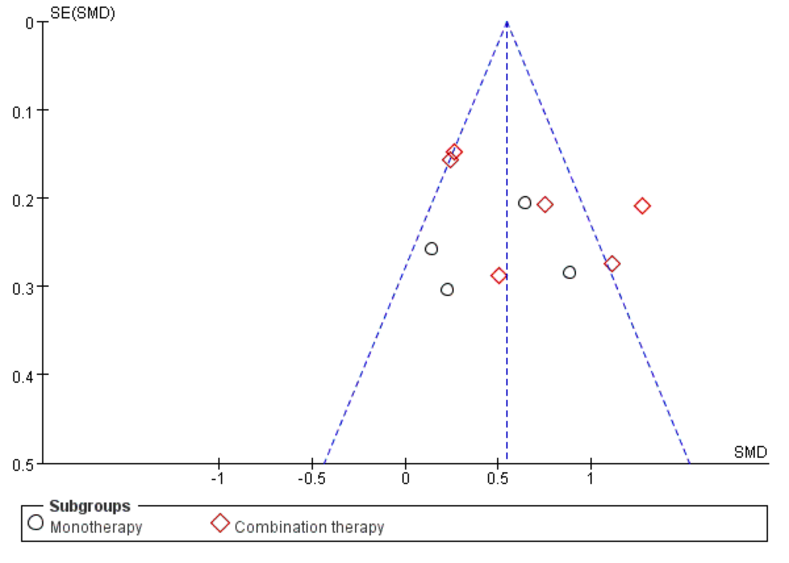

Supplement: S10 Fig — (TIF) [file pone.0313074.s012.tif]
