# Supplementary material for: Efficacy of platelet-rich plasma in the treatment of erectile dysfunction: A meta-analysis of controlled and single-arm trials
Source: PLoS One. 2024 Nov 14;19(11):e0313074. doi: 10.1371/journal.pone.0313074 (PMC11563399; doi:10.1371/journal.pone.0313074)
Supplement: S1 Table — (DOCX) [file pone.0313074.s013.docx]

| **Research** | **RCT** | | **Single-arm trials** | | **DOI or Title** |
| --- | --- | --- | --- | --- | --- |
|  | **Included** | **The reason for exclusion** | **Included** | **The reason for exclusion** |  |
|  |  |  |  |  |  |
| Barone 2022 | Yes | - | - | - | DOI: 10.1016/S2666-1683(22)01007-2 |
| Boyuk, 2022 | Yes | - | - | - | DOI: 10.1016/S2666-1683(22)00143-4 |
| Epifanova, 2024 | Yes | - | - | - | DOI: 10.1016/S0302-2838(24)00269-0 |
| Geyik, 2021 | Yes | - | - | - | DOI: 10.1111/and.14197 |
| Khalef, 2023 | Yes | - | - | - | DOI: 10.37506/ijfmt.v17i1.18906 |
| Ledesma, 2023 | Yes | - | - | - | DOI: 10.1097/JU.0000000000003360.12 |
| Masterson, 2023 | Yes | - | - | - | DOI: 10.1097/JU.0000000000003481 |
| Poulios, 2021 | Yes | - | - | - | DOI: 10.1016/j.jsxm.2021.03.008 |
| Ruffo, 2019 | Yes | - | - | - | DOI: 10.1016/S1569-9056(19)31175-3 |
| Ruffo, 2020 | Yes | - | - | - | DOI: 10.1016/j.jsxm.2020.04.048 |
| Shaher, 2023 | Yes | - | - | - | DOI: 10.1016/j.urology.2023.01.028 |
| Achraf, 2023 | - | - | Yes | - | DOI: 10.1080/2090598X.2022.2135284 |
| Banno, 2017 | - | - | Yes | - | The efficacy of platelet-rich plasma (PRP) as a supplemental therapy for the treatment of erectile dysfunction (ED): Initial outcomes |
| Epifanova, 2019 | - | - | Yes | - | DOI: 10.1111/bju.14730 |
| Epifanova, 2020 | - | - | Yes | - | DOI: 10.1016/j.jsxm.2020.04.170 |
| Francomano, 2023 | - | - | Yes | - | DOI: 10.23736/S2724-6507.23.04060-5 |
| Schirmann, 2022 | - | - | Yes | - | DOI: 10.1016/j.purol.2022.05.002 |
| Schirmann, 2022 | - | - | Yes | - | DOI: 10.1016/j.purol.2022.05.004 |
| Taş, 2021 | - | - | Yes | - | DOI: 10.1016/j.esxm.2020.100313 |
| Wong, 2021 | - | - | Yes | - | DOI: 10.4103/UROS.UROS_22_21 |
| Zaghloul, 2021 | - | - | Yes | - | DOI: 10.1111/and.14162 |
| Shahinyan, 2022 | No | Non clinical study | No | Non clinical study | DOI: 10.1001/jamanetworkopen.2022.14187 |
| Ge, 2023 | No | Non clinical study | No | Non clinical study | DOI: 10.1016/j.lfs.2023.121767 |
| Zavala, 2023 | No | Non clinical study | No | Non clinical study | DOI: https://doi.org/10.1055/s-0040-1721562 |
| Lu, 2021 | No | Non clinical study | No | Non clinical study | DOI: 10.1111/cns.13640 |
| Wu, 2021 | No | Non clinical study | No | Non clinical study | DOI: 10.1016/j.jsxm.2020.12.016 |
| Towe, 2020 | No | Non clinical study | No | Non clinical study | DOI: 10.1016/S2666-1683(20)33338-3 |
| Towe, 2021 | No | Non clinical study | No | Non clinical study | DOI: 10.1038/s41443-020-0263-3 |
| Wu, 2022 | No | Non clinical study | No | Non clinical study | DOI: 10.1016/j.jfma.2021.01.019 |
| Ding, 2008 | No | Non clinical study | No | Non clinical study | Effect of platelet rich plasma on the regeneration of cavernous nerve: Experiment with rats |
| Ding, 2009 | No | Non clinical study | No | Non clinical study | DOI: 10.1038/aja.2008.37 |
| Liao, 2018 | No | Non clinical study | No | Non clinical study | Effects of platelet rich plasma on improving erectile dysfunction in streptozotocin-induced diabetic rats |
| Liao, 2023 | No | Non clinical study | No | Non clinical study | DOI: 10.1016/j.biopha.2023.114499 |
| Huang, 2021 | No | Non clinical study | No | Non clinical study | DOI: https://dx.doi.org/10.1016/j.esxm.2020.100317 |
| Tai, 2023 | No | Non clinical study | No | Non clinical study | DOI: 10.1096/fj.202201443R |
| Liao, 2022 | No | Non clinical study | No | Non clinical study | DOI: https://dx.doi.org/10.3390/ijms23063017 |
| Wu, 2012 | No | Non clinical study | No | Non clinical study | DOI: https://doi.org/10.1111/j.1743-6109.2012.02881.x |
| Wu, 2016 | No | Non clinical study | No | Non clinical study | DOI: 10.1002/term.1806 |
| Shahinyan, 2021 | No | Non clinical study | No | Non clinical study | DOI: 10.1016/j.jsxm.2021.01.064 |
| Ghomeshi, 2024 | No | Non clinical study | No | Non clinical study | DOI: 10.1093/jsxmed/qdae001.227 |
| Best, 2023 | No | Non clinical study | No | Non clinical study | DOI: 10.1097/JU.0000000000003356.04 |
| Weinberger, 2021 | No | Non clinical study | No | Non clinical study | DOI: 10.1016/j.jsxm.2021.01.068 |
| Alkhayal, 2019 | No | Incomplete experimental data | No | Incomplete experimental data | DOI: 10.1016/j.jsxm.2019.03.228 |
| Angulo, 2023 | No | Incomplete experimental data | No | Incomplete experimental data | DOI: 10.1093/jsxmed/qdad062.007 |
| Epifanova, 2020 | No | Incomplete experimental data | No | Incomplete experimental data | DOI: 10.1016/S2666-1683(20)33096-2 |
| Fliatouras, 2023 | No | Incomplete experimental data | No | Incomplete experimental data | DOI: 10.1093/jsxmed/qdad062.108 |
| Mayer, 2024 | No | Incomplete experimental data | No | Incomplete experimental data | DOI: 10.1093/jsxmed/qdae018.021 |
| Virag, 2016 | No | Incomplete experimental data | No | Incomplete experimental data | Ultrasound guided treatment of peyronie's disease with plasma rich platelets (PRP) and hyaluronic acid (HA) |
| Virag, 2017 | No | Incomplete experimental data | No | Incomplete experimental data | Improves peyronie's disease: A case control study of 75 cases plasma rich platelets and hyaluronic acid |
| Zugail, 2024 | No | Incomplete experimental data | No | Incomplete experimental data | DOI: 10.1038/s41443-023-00744-y |
| Fernandez-Pascual, 2020 | No | Incomplete experimental data | No | Incomplete experimental data | DOI: 10.1016/j.jsxm.2020.04.055 |
| Ledesma, 2024 | No | Incomplete experimental data | No | Incomplete experimental data | DOI: 10.1093/jsxmed/qdae001.142 |
| Ragheb, 2024 | No | Incomplete experimental data | No | Incomplete experimental data | DOI: 10.1093/jsxmed/qdae002.014 |
| Ruffo, 2018 | No | Incomplete experimental data | No | Incomplete experimental data | DOI: 10.1016/j.jsxm.2018.04.435 |
| Saltzman, 2023 | No | Incomplete experimental data | No | Incomplete experimental data | DOI: 10.1016/j.euf.2022.09.015 |
| Chu, 2023 | No | No research result | No | No research result | DOI: 10.1016/j.euf.2022.08.017 |
| Epifanova, 2022 | No | No research result | No | No research result | Clinical trial evaluating platelet-rich plasma and extracorporeal shock wave therapy using in erectile dysfunction treatment |
| Raaia, 2019 | No | No research result | No | No research result | DOI: 10.37506/v10/i12/2019/ijphrd/192414 |
| Zasieda, 2020 | No | No research result | No | No research result | DOI: 10.1016/j.jsxm.2020.04.120 |
| Zucker, 2023 | No | No research result | No | No research result | EXAMINING AND SAFETY AND SIDE EFFECTS OF PLATELET-RICH PLASMA VERSUS PLACEBO IN A PHASE III CLINICAL TRIAL IN THE MANAGEMENT OF ERECTILE DYSFUNCTION |
| Alshuaibi, 2024 | No | Wrong study design | No | Wrong study design | DOI: https://doi.org/10.1016/j.purol.2023.09.013 |
| Channapppa, 2016 | No | Wrong study design | No | Wrong study design | DOI: 10.1016/j.jsxm.2016.03.037 |
| Hilton, 2017 | No | Wrong study design | No | Wrong study design | 10.1002/central/CN-02113368/full |
| Ledesma, 2024 | No | Wrong study design | No | Wrong study design | DOI: 10.1038/s41443-024-00844-3 |
| Lu, 2020 | No | Wrong study design | No | Wrong study design | DOI: 10.1016/j.jsxm.2019.11.145 |
| Ruffo, 2023 | No | Wrong study design | No | Wrong study design | TRIMODAL THERAPY FOR ERECTILE DYSFUNCTION: COMBINATION OF LOW-INTENSITY SHOCK WAVES, PLATELET RICH PLASMA (PRP) INJECTIONS AND VACUUM ERECTILE DEVICE PHYSIOTHERAPY |
| Thomas, 2023 | No | Wrong study design | No | Wrong study design | DOI: 10.1016/j.urology.2023.01.061 |
| Virag, 2017 | No | Wrong study design | No | Wrong study design | DOI: 10.1016/j.jsxm.2017.03.112 |
| Yiou, 2017 | No | Wrong study design | No | Wrong study design | DOI: 10.3233/BME-171627 |
| Zaghloul, 2022 | No | Wrong study design | No | Wrong study design | DOI: 10.1111/and.14421 |
| Epifanova, 2018 | No | Wrong study design | No | Wrong study design | DOI: 10.1016/j.jsxm.2018.04.251 |
| Fernandez Pascual, 2024 | No | Wrong study design | No | Wrong study design | DOI: 10.1016/S0302-2838(24)01662-2 |
| Ctri, 2023 | No | Ongoing clinical trails | No | Ongoing clinical trails | 10.1002/central/CN-02604308/full |
| Euctr, 2020 | No | Ongoing clinical trails | No | Ongoing clinical trails | 10.1002/central/CN-02169243/full |
| Euctr, 2022 | No | Ongoing clinical trails | No | Ongoing clinical trails | 10.1002/central/CN-02597538/full |
| Irct20230609058433N, 2023 | No | Ongoing clinical trails | No | Ongoing clinical trails | 10.1002/central/CN-02586448/full |
| Nct, 2020 | No | Ongoing clinical trails | No | Ongoing clinical trails | 10.1002/central/CN-02093706/full |
| Nct, 2020 | No | Ongoing clinical trails | No | Ongoing clinical trails | 10.1002/central/CN-02145890/full |
| Nct, 2021 | No | Ongoing clinical trails | No | Ongoing clinical trails | 10.1002/central/CN-02331646/full |
| Nct, 2023 | No | Ongoing clinical trails | No | Ongoing clinical trails | 10.1002/central/CN-02538430/full |
| Nct, 2024 | No | Ongoing clinical trails | No | Ongoing clinical trails | 10.1002/central/CN-02680380/full |
| Abate, 2021 | No | Non-English literature | No | Non-English literature | https://doi.org/10.1016/j.rcot.2020.11.019 |
| Boucher, 1996 | No | Non-English literature | No | Non-English literature | https://doi.org/10.1016/0929-693X(96)82159-2 |
| Escobar, 2015 | No | Non-English literature | No | Non-English literature | https://doi.org/10.1016/j.rccot.2016.03.003 |
| Alkandari, 2021 | No | Letter and review | No | Letter and review | DOI: 10.1016/j.jsxm.2021.01.123 |
| Alkandari, 2022 | No | Letter and review | No | Letter and review | DOI: 10.1016/j.sxmr.2020.12.004 |
| Anastasiadis, 2022 | No | Letter and review | No | Letter and review | https://dx.doi.org/10.3389/frph.2022.944765 |
| Atmoko, 2024 | No | Letter and review | No | Letter and review | DOI: 10.1093/jsxmed/qdae002.028 |
| Bole, 2023 | No | Letter and review | No | Letter and review | DOI: 10.1016/j.urology.2023.01.062 |
| Britt, 2021 | No | Letter and review | No | Letter and review | https://dx.doi.org/10.5489/cuaj.6947 |
| Chalyj, 2015 | No | Letter and review | No | Letter and review | THE EFFECTIVENESS OF INTRACAVERNOUS AUTOLOGOUS PLATELET-RICH PLASMA IN THE TREATMENT OF ERECTILE DYSFUNCTION |
| Deabes, 2024 | No | Letter and review | No | Letter and review | DOI: 10.1093/sxmrev/qeae018 |
| Duymus, 2016 | No | Letter and review | No | Letter and review | DOI: 10.1177/1602400240 |
| Epifanova, 2020 | No | Letter and review | No | Letter and review | DOI: 10.1016/j.sxmr.2019.02.002 |
| Fazekas, 2023 | No | Letter and review | No | Letter and review | DOI: 10.1093/sxmrev/qead032 |
| Fode, 2024 | No | Letter and review | No | Letter and review | DOI: 10.1016/j.eururo.2023.08.018 |
| Ganijee, 2023 | No | Letter and review | No | Letter and review | DOI: 10.1016/j.urology.2023.08.010 |
| Grandez-Urbina, 2019 | No | Letter and review | No | Letter and review | DOI: 10.1016/j.acuro.2018.05.008 |
| Israeli, 2022 | No | Letter and review | No | Letter and review | DOI: 10.1038/s41443-021-00482-z |
| Jiang, 2014 | No | Letter and review | No | Letter and review | DOI: 10.13481/j.1671-587x.20140345 |
| Kalyvianakis, 2023 | No | Letter and review | No | Letter and review | DOI: 10.1097/JU.0000000000003684 |
| Khamedov, 2022 | No | Letter and review | No | Letter and review | DOI: 10.21886/2308-6424-2022-10-1-121-127 |
| Masterson, 2023 | No | Letter and review | No | Letter and review | DOI: 10.1097/JU.0000000000003685 |
| Matz, 2022 | No | Letter and review | No | Letter and review | DOI: 10.1016/j.sxmr.2020.07.001 |
| Muchedzi, 2018 | No | Letter and review | No | Letter and review | DOI: 10.1016/j.surge.2017.08.004 |
| Ngo, 2021 | No | Letter and review | No | Letter and review | DOI: 10.15419/BMRAT.V8I10.698 |
| Obolenskiy, 2017 | No | Letter and review | No | Letter and review | https://doi.org/10.1016/j.wndm.2017.09.001 |
| Panunzio, 2023 | No | Letter and review | No | Letter and review | DOI: 10.1038/s41443-023-00798-y |
| Panunzio, 2024 | No | Letter and review | No | Letter and review | DOI: 10.1038/s41443-024-00878-7 |
| Popov, 2023 | No | Letter and review | No | Letter and review | DOI: 10.21886/2308-6424-2023-11-1-134-142 |
| Poulios, 2023 | No | Letter and review | No | Letter and review | DOI: 10.1093/sxmrev/qead027 |
| Şahin, 2024 | No | Letter and review | No | Letter and review | DOI: 10.1038/s41443-023-00806-1 |
| Savira, 2024 | No | Letter and review | No | Letter and review | DOI: 10.1093/jsxmed/qdae002.106 |
| Scott, 2019 | No | Letter and review | No | Letter and review | DOI: 10.1016/j.sxmr.2018.12.006 |
| Scott, 2018 | No | Letter and review | No | Letter and review | DOI: 10.1111/bju.14117 |
| Tchetina, 2023 | No | Letter and review | No | Letter and review | DOI: 10.1186/s13018-023-03629-4 |
| Thomas, 2023 | No | Letter and review | No | Letter and review | DOI: 10.1016/j.urology.2023.01.063 |
| Victor, 2024 | No | Letter and review | No | Letter and review | DOI: 10.1038/s41443-024-00850-5 |
| Wroblewski, 2010 | No | Letter and review | No | Letter and review | https://doi.org/10.1053/j.oto.2009.10.006 |
| Yáñez-Castillo, 2022 | No | Letter and review | No | Letter and review | DOI: 10.56434/j.arch.esp.urol.20227508.98 |
| Zlotnicki, 2016 | No | Letter and review | No | Letter and review | DOI: https://doi.org/10.1053/j.oto.2015.11.002 |
| Chen, 2018 | No | Letter and review | No | Letter and review | DOI: 10.13481/j.1671-587x.20180133 |
| Chung, 2019 | No | Letter and review | No | Letter and review | DOI: 10.3390/medsci7090091 |
| Chung, 2021 | No | Letter and review | No | Letter and review | DOI: 10.1177/17562872211026421 |
| Davidson, 2014 | No | Letter and review | No | Letter and review | DOI: 10.2217/FNL.13.74 |
| Foti, 2024 | No | Letter and review | No | Letter and review | DOI: 10.1097/JSA.0000000000000399 |
| Green, 2018 | No | Letter and review | No | Letter and review | DOI: 10.1016/B978-0-444-63945-5.00019-2 |
| Irwin, 2019 | No | Letter and review | No | Letter and review | DOI: 10.1016/j.pop.2019.02.006 |
| Jiao, 2018 | No | Letter and review | No | Letter and review | DOI: 10.13481/j.1671-587x.20180241 |
| Abazari, 2019 | No | Not related to the topic | No | Not related to the topic | DOI: https://doi.org/10.1016/j.gene.2019.144096 |
| Abdel Hafez, 2021 | No | Not related to the topic | No | Not related to the topic | DOI: https://doi.org/10.1016/j.intimp.2021.107814 |
| Abdelbary, 2018 | No | Not related to the topic | No | Not related to the topic | DOI: https://doi.org/10.1016/j.ejrnm.2018.07.019 |
| Abdullah, 2019 | No | Not related to the topic | No | Not related to the topic | DOI: https://doi.org/10.1016/j.amsu.2018.11.009 |
| Abrar, 2022 | No | Not related to the topic | No | Not related to the topic | DOI: https://doi.org/10.1016/j.ortho.2022.100694 |
| AbuBakr, 2022 | No | Not related to the topic | No | Not related to the topic | DOI: https://doi.org/10.1016/j.heliyon.2022.e10857 |
| Ackermann, 2015 | No | Not related to the topic | No | Not related to the topic | DOI: https://doi.org/10.1016/j.mvr.2015.09.001 |
| Aftab, 2020 | No | Not related to the topic | No | Not related to the topic | DOI: https://doi.org/10.1016/j.ajoms.2020.03.008 |
| Aggarwal, 2021 | No | Not related to the topic | No | Not related to the topic | DOI: 10.1016/j.arth.2020.07.010 |
| Aggarwal, 2014 | No | Not related to the topic | No | Not related to the topic | DOI: 10.1007/s00264-013-2136-6 |
| Aghaloo, 2002 | No | Not related to the topic | No | Not related to the topic | DOI: https://doi.org/10.1053/joms.2002.34994 |
| Ahmed, 2017 | No | Not related to the topic | No | Not related to the topic | DOI: https://doi.org/10.1016/j.avsg.2016.04.023 |
| Akbulut, 2019 | No | Not related to the topic | No | Not related to the topic | DOI: https://doi.org/10.1016/j.ajodo.2018.03.015 |
| Alaa, 2023 | No | Not related to the topic | No | Not related to the topic | DOI: https://doi.org/10.1016/j.ortho.2022.100715 |
| Albano, 2017 | No | Not related to the topic | No | Not related to the topic | DOI: 10.1016/j.ejrad.2017.08.006 |
| Alcay, 2021 | No | Not related to the topic | No | Not related to the topic | DOI: https://doi.org/10.1016/j.cryobiol.2021.09.012 |
| Alessio-Mazzola, 2023 | No | Not related to the topic | No | Not related to the topic | DOI: https://doi.org/10.1053/j.jfas.2022.10.005 |
| Ali, 2018 | No | Not related to the topic | No | Not related to the topic | DOI: 10.15557/JoU.2018.0048 |
| Alio, 2007 | No | Not related to the topic | No | Not related to the topic | DOI: https://doi.org/10.1016/j.ophtha.2006.10.044 |
| Alkhayal, 2018 | No | Not related to the topic | No | Not related to the topic | DOI: 10.1016/j.jsxm.2018.04.283 |
| Alkhayal, 2019 | No | Not related to the topic | No | Not related to the topic | DOI: 10.1016/j.jsxm.2019.03.228 |
| Allahveisi, 2020 | No | Not related to the topic | No | Not related to the topic | DOI: https://doi.org/10.1016/j.heliyon.2020.e03577 |
| Almasry, 2015 | No | Not related to the topic | No | Not related to the topic | DOI: https://doi.org/10.1016/j.aanat.2014.10.006 |
| Althaus, 2019 | No | Not related to the topic | No | Not related to the topic | DOI: https://doi.org/10.1016/j.thromres.2019.05.016 |
| Arif, 2023 | No | Not related to the topic | No | Not related to the topic | DOI: https://doi.org/10.1016/j.kjs.2023.01.007 |
| Arpornmaeklong, 2004 | No | Not related to the topic | No | Not related to the topic | DOI: https://doi.org/10.1054/ijom.2003.0492 |
| Arumugam, 2021 | No | Not related to the topic | No | Not related to the topic | DOI: 10.1007/s43465-020-00349-3 |
| Asadpour, 2022 | No | Not related to the topic | No | Not related to the topic | DOI: https://doi.org/10.1016/j.joms.2022.05.002 |
| Atashi, 2019 | No | Not related to the topic | No | Not related to the topic | DOI: https://doi.org/10.1016/j.bjps.2018.12.039 |
| Atwa, 2019 | No | Not related to the topic | No | Not related to the topic | DOI: https://doi.org/10.1016/j.ejr.2018.07.008 |
| Ayala-Montes de Oca, 2016 | No | Not related to the topic | No | Not related to the topic | DOI: https://doi.org/10.1016/j.hgmx.2016.05.006 |
| Aydın, 2020 | No | Not related to the topic | No | Not related to the topic | DOI: https://doi.org/10.1016/j.urology.2020.03.025 |
| Azcárate, 2014 | No | Not related to the topic | No | Not related to the topic | DOI: https://doi.org/10.1016/S0020-1383(14)70008-7 |
| Baba, 2019 | No | Not related to the topic | No | Not related to the topic | DOI: https://doi.org/10.1016/j.jcms.2019.01.020 |
| Barba-Recreo, 2015 | No | Not related to the topic | No | Not related to the topic | DOI: https://doi.org/10.1016/j.jcms.2015.04.026 |
| Barber, 2016 | No | Not related to the topic | No | Not related to the topic | DOI: https://doi.org/10.1016/j.arthro.2015.11.020 |
| Barber, 2011 | No | Not related to the topic | No | Not related to the topic | DOI: https://doi.org/10.1016/j.arthro.2011.06.010 |
| Baria, 2024 | No | Not related to the topic | No | Not related to the topic | DOI: 10.1177/23259671241233916 |
| Baria, 2022 | No | Not related to the topic | No | Not related to the topic | DOI: 10.1177/23259671221120678 |
| Barman, 2023 | No | Not related to the topic | No | Not related to the topic | DOI: https://doi.org/10.1016/j.injury.2022.11.036 |
| Barman, 2022 | No | Not related to the topic | No | Not related to the topic | DOI: https://doi.org/10.1016/j.injury.2022.01.012 |
| Batista, 2011 | No | Not related to the topic | No | Not related to the topic | DOI: https://doi.org/10.1590/S1807-593220110007000018 |
| Batstone, 2012 | No | Not related to the topic | No | Not related to the topic | DOI: https://doi.org/10.1016/j.ijom.2011.06.018 |
| Bava, 2011 | No | Not related to the topic | No | Not related to the topic | DOI: 10.3810/psm.2011.09.1925 |
| Bayat, 2020 | No | Not related to the topic | No | Not related to the topic | DOI: https://doi.org/10.1016/j.brainresbull.2020.08.033 |
| Baz, 2017 | No | Not related to the topic | No | Not related to the topic | DOI: https://doi.org/10.1016/j.ejrnm.2016.12.004 |
| Becerra-Bayona, 2022 | No | Not related to the topic | No | Not related to the topic | DOI: https://doi.org/10.1016/j.biologicals.2021.11.001 |
| Beck, 2012 | No | Not related to the topic | No | Not related to the topic | DOI: 10.1177/0363546512453300 |
| Beek, 2020 | No | Not related to the topic | No | Not related to the topic | DOI: https://doi.org/10.1016/j.nurpra.2019.08.018 |
| Bharathi Mohan, 2020 | No | Not related to the topic | No | Not related to the topic | DOI: 10.4103/jcas.Jcas_112_19 |
| Bhatt, 2023 | No | Not related to the topic | No | Not related to the topic | DOI: https://doi.org/10.1016/j.jvoice.2020.12.040 |
| Biedermann, 2024 | No | Not related to the topic | No | Not related to the topic | DOI: https://doi.org/10.1016/j.arthro.2024.04.015 |
| Bielecki, 2012 | No | Not related to the topic | No | Not related to the topic | DOI: https://doi.org/10.1016/j.transci.2012.06.017 |
| Bini, 1994 | No | Not related to the topic | No | Not related to the topic | DOI: https://doi.org/10.1016/0049-3848(94)90185-6 |
| Binici, 2024 | No | Not related to the topic | No | Not related to the topic | DOI: 10.1007/s00418-024-02267-z |
| Błaszczyk, 2018 | No | Not related to the topic | No | Not related to the topic | DOI: 10.1155/2018/4120471 |
| Bocanegra-Pérez, 2012 | No | Not related to the topic | No | Not related to the topic | DOI: https://doi.org/10.1016/j.ijom.2012.04.020 |
| Boden, 2019 | No | Not related to the topic | No | Not related to the topic | DOI: https://doi.org/10.1016/j.jse.2018.08.032 |
| Borda, 1980 | No | Not related to the topic | No | Not related to the topic | DOI: https://doi.org/10.1016/0090-6980(80)90124-0 |
| Borhani-Haghighi, 2019 | No | Not related to the topic | No | Not related to the topic | DOI: https://doi.org/10.1016/j.jneuroim.2019.04.018 |
| Bosch, 2011 | No | Not related to the topic | No | Not related to the topic | DOI: https://doi.org/10.1016/j.tvjl.2009.10.014 |
| Bostancı, 2022 | No | Not related to the topic | No | Not related to the topic | DOI: 10.1111/jog.15232 |
| Branch, 2023 | No | Not related to the topic | No | Not related to the topic | DOI: https://doi.org/10.1016/j.jcjp.2023.100129 |
| Branch, 2021 | No | Not related to the topic | No | Not related to the topic | DOI: 10.1016/j.arthro.2020.09.035 |
| Breton, 2022 | No | Not related to the topic | No | Not related to the topic | DOI: https://doi.org/10.1016/j.diii.2021.10.008 |
| Brewer, 2022 | No | Not related to the topic | No | Not related to the topic | DOI: 10.1007/s00268-022-06711-w |
| Brinkman, 2023 | No | Not related to the topic | No | Not related to the topic | DOI: https://doi.org/10.1016/j.asmr.2023.04.026 |
| Brkljac, 2019 | No | Not related to the topic | No | Not related to the topic | DOI: https://doi.org/10.1016/j.jor.2019.08.023 |
| Brkljac, 2015 | No | Not related to the topic | No | Not related to the topic | DOI: https://doi.org/10.1016/j.jor.2015.10.018 |
| Bugarin, 2022 | No | Not related to the topic | No | Not related to the topic | DOI: 10.1177/23259671221093074 |
| Butterfield, 2005 | No | Not related to the topic | No | Not related to the topic | DOI: https://doi.org/10.1016/j.joms.2004.07.017 |
| Byvaltsev, 2019 | No | Not related to the topic | No | Not related to the topic | DOI: 10.17116/jnevro201911911127 |
| Cabbar, 2011 | No | Not related to the topic | No | Not related to the topic | DOI: https://doi.org/10.1016/j.joms.2011.03.040 |
| Cabrera-Ramírez, 2017 | No | Not related to the topic | No | Not related to the topic | DOI: https://doi.org/10.1016/j.adengl.2017.07.002 |
| Cai, 2015 | No | Not related to the topic | No | Not related to the topic | DOI: https://doi.org/10.1016/j.jse.2015.07.035 |
| Calisir, 2020 | No | Not related to the topic | No | Not related to the topic | DOI: https://doi.org/10.1016/j.jss.2020.06.037 |
| Calori, 2008 | No | Not related to the topic | No | Not related to the topic | DOI: https://doi.org/10.1016/j.injury.2008.08.011 |
| Cao, 2023 | No | Not related to the topic | No | Not related to the topic | DOI: https://doi.org/10.1016/j.isci.2023.108236 |
| Cao, 2022 | No | Not related to the topic | No | Not related to the topic | DOI: https://doi.org/10.1016/j.jot.2022.02.002 |
| Carlier, 2021 | No | Not related to the topic | No | Not related to the topic | DOI: https://doi.org/10.1016/j.otsr.2020.03.016 |
| Carney, 2020 | No | Not related to the topic | No | Not related to the topic | DOI: https://doi.org/10.1016/j.reth.2020.07.004 |
| Carter, 2003 | No | Not related to the topic | No | Not related to the topic | DOI: https://doi.org/10.1016/S0014-4800(03)00017-0 |
| Casati, 2007 | No | Not related to the topic | No | Not related to the topic | DOI: https://doi.org/10.1016/j.ijom.2006.06.004 |
| Castillo, 2020 | No | Not related to the topic | No | Not related to the topic | DOI: https://doi.org/10.1016/j.jcol.2020.05.002 |
| Castro, 2014 | No | Not related to the topic | No | Not related to the topic | DOI: https://doi.org/10.1016/j.rvsc.2013.12.005 |
| Castro, 2019 | No | Not related to the topic | No | Not related to the topic | DOI: https://doi.org/10.1016/j.brainresbull.2019.07.024 |
| Cavendish, 2020 | No | Not related to the topic | No | Not related to the topic | DOI: https://doi.org/10.1016/j.jse.2020.01.084 |
| Célio-Mariano, 2012 | No | Not related to the topic | No | Not related to the topic | DOI: https://doi.org/10.1016/j.joms.2011.03.028 |
| Cen, 2024 | No | Not related to the topic | No | Not related to the topic | DOI: https://doi.org/10.1016/j.otsr.2022.103417 |
| Cerci, 2015 | No | Not related to the topic | No | Not related to the topic | DOI: https://doi.org/10.1016/j.jcms.2015.06.040 |
| Cerciello, 2013 | No | Not related to the topic | No | Not related to the topic | DOI: https://doi.org/10.1053/j.oto.2013.07.001 |
| Cervelli, 2014 | No | Not related to the topic | No | Not related to the topic | DOI: 10.1155/2014/760709 |
| Çetiner, 2018 | No | Not related to the topic | No | Not related to the topic | DOI: https://doi.org/10.1016/j.jds.2017.11.002 |
| Çetinkaya, 2019 | No | Not related to the topic | No | Not related to the topic | DOI: 10.1007/s00068-018-0957-0 |
| Cetinkaya, 2018 | No | Not related to the topic | No | Not related to the topic | DOI: 10.1007/s00068-017-0852-0 |
| Chai, 2019 | No | Not related to the topic | No | Not related to the topic | DOI: 10.1016/j.joen.2019.04.002 |
| Chan, 2022 | No | Not related to the topic | No | Not related to the topic | DOI: https://doi.org/10.1016/j.jos.2020.12.012 |
| Chan, 2023 | No | Not related to the topic | No | Not related to the topic | DOI: 10.1177/23259671231187894 |
| Chandak, 2022 | No | Not related to the topic | No | Not related to the topic | DOI: https://doi.org/10.1016/j.ajodo.2022.03.013 |
| Chang, 2023 | No | Not related to the topic | No | Not related to the topic | DOI: https://doi.org/10.1016/j.jri.2023.103796 |
| Charousset, 2014 | No | Not related to the topic | No | Not related to the topic | DOI: https://doi.org/10.1016/j.arthro.2013.12.018 |
| Chen, 2024 | No | Not related to the topic | No | Not related to the topic | DOI: https://doi.org/10.1016/j.jss.2023.08.029 |
| Chen, 2021 | No | Not related to the topic | No | Not related to the topic | DOI: https://doi.org/10.1016/j.apmr.2020.12.025 |
| Chen, 2023 | No | Not related to the topic | No | Not related to the topic | DOI: https://doi.org/10.1016/j.knee.2023.10.005 |
| Cheng, 2018 | No | Not related to the topic | No | Not related to the topic | DOI: 10.1016/j.ijpharm.2018.06.020 |
| Chiang, 2014 | No | Not related to the topic | No | Not related to the topic | DOI: https://doi.org/10.1016/j.jecm.2014.06.007 |
| Chiavaras, 2014 | No | Not related to the topic | No | Not related to the topic | DOI: https://doi.org/10.1016/j.acra.2014.05.003 |
| Chieregato, 2011 | No | Not related to the topic | No | Not related to the topic | DOI: https://doi.org/10.3109/14653249.2011.583232 |
| Chignard, 1978 | No | Not related to the topic | No | Not related to the topic | DOI: https://doi.org/10.1016/0006-291X(78)91190-7 |
| Chikazu, 2005 | No | Not related to the topic | No | Not related to the topic | DOI: https://doi.org/10.1016/S0915-6992(05)80029-9 |
| Childers, 2018 | No | Not related to the topic | No | Not related to the topic | DOI: https://doi.org/10.1021/acs.biomac.8b00725 |
| Childers, 2016 | No | Not related to the topic | No | Not related to the topic | DOI: https://doi.org/10.1021/acs.biomac.6b01195 |
| Cho, 2011 | No | Not related to the topic | No | Not related to the topic | DOI: https://doi.org/10.1016/j.bjps.2010.08.014 |
| Choi, 2004 | No | Not related to the topic | No | Not related to the topic | DOI: https://doi.org/10.1054/ijom.2003.0466 |
| Chouhan, 2019 | No | Not related to the topic | No | Not related to the topic | DOI: 10.1177/0363546519856605 |
| Chrysanthopoulou, 2017 | No | Not related to the topic | No | Not related to the topic | DOI: https://doi.org/10.1016/j.mehy.2017.05.018 |
| Chu, 2022 | No | Not related to the topic | No | Not related to the topic | DOI: 10.1001/jama.2021.24745 |
| Cohen, 2019 | No | Not related to the topic | No | Not related to the topic | Laser-assisted drug del ivery for the treatment of androgenetic alopecia: Ablative laser fractional photothermolysis to enhance cutaneous topical delivery of platelet-rich plasma - with or without concurrent bimatoprost and/or minoxidil |
| Cohen, 2021 | No | Not related to the topic | No | Not related to the topic | DOI: https://doi.org/10.1016/j.arth.2021.05.040 |
| Coller, 1976 | No | Not related to the topic | No | Not related to the topic | DOI: https://doi.org/10.1182/blood.V47.5.841.841 |
| Coşkun, 2022 | No | Not related to the topic | No | Not related to the topic | DOI: 10.7759/cureus.24500 |
| Coskun, 2019 | No | Not related to the topic | No | Not related to the topic | DOI: https://doi.org/10.1016/j.jcms.2018.12.004 |
| Costa, 2023 | No | Not related to the topic | No | Not related to the topic | DOI: 10.3390/biomedicines12010006 |
| Cunha, 2014 | No | Not related to the topic | No | Not related to the topic | DOI: https://doi.org/10.1016/j.transproceed.2014.05.063 |
| Czakai, 2017 | No | Not related to the topic | No | Not related to the topic | DOI: https://doi.org/10.1016/j.ijmm.2016.11.010 |
| Deal, 2017 | No | Not related to the topic | No | Not related to the topic | DOI: 10.1177/2325967117738238 |
| Debs, 2021 | No | Not related to the topic | No | Not related to the topic | DOI: https://doi.org/10.1016/j.jss.2021.06.066 |
| DeChellis, 2011 | No | Not related to the topic | No | Not related to the topic | DOI: https://doi.org/10.1053/j.trap.2011.05.002 |
| Delgado, 2021 | No | Not related to the topic | No | Not related to the topic | DOI: 10.3390/ijms22041725 |
| DelRossi, 1990 | No | Not related to the topic | No | Not related to the topic | DOI: https://doi.org/10.1016/S0022-5223(19)35569-2 |
| Desai, 2023 | No | Not related to the topic | No | Not related to the topic | DOI: 10.4103/ccd.ccd_419_21 |
| Devereaux, 2018 | No | Not related to the topic | No | Not related to the topic | DOI: https://doi.org/10.1016/j.maturitas.2018.09.001 |
| Dhamija, 2020 | No | Not related to the topic | No | Not related to the topic | DOI: https://doi.org/10.1016/j.joen.2020.06.004 |
| Dhillon, 2015 | No | Not related to the topic | No | Not related to the topic | DOI: 10.11138/mltj/2015.5.3.156 |
| Diaz-Gomez, 2014 | No | Not related to the topic | No | Not related to the topic | DOI: https://doi.org/10.1016/j.msec.2014.03.065 |
| Dietrich, 2017 | No | Not related to the topic | No | Not related to the topic | DOI: 10.1080/17453674.2017.1293447 |
| Ding, 2024 | No | Not related to the topic | No | Not related to the topic | DOI: 10.1002/adhm.202303192 |
| Ding, 2023 | No | Not related to the topic | No | Not related to the topic | DOI: 10.1007/s00264-023-05773-2 |
| Dizdar, 2024 | No | Not related to the topic | No | Not related to the topic | DOI: 10.1016/j.anl.2023.12.003 |
| Doerger, 1988 | No | Not related to the topic | No | Not related to the topic | DOI: https://doi.org/10.1016/0049-3848(88)90324-6 |
| Eda, 2017 | No | Not related to the topic | No | Not related to the topic | DOI: https://doi.org/10.1016/j.ajoms.2017.06.011 |
| Eftekhar, 2018 | No | Not related to the topic | No | Not related to the topic | DOI: https://doi.org/10.1016/j.tjog.2018.10.007 |
| Eissa, 2022 | No | Not related to the topic | No | Not related to the topic | DOI: https://doi.org/10.1016/j.fawpar.2022.e00180 |
| Ekelem, 2020 | No | Not related to the topic | No | Not related to the topic | DOI: https://doi.org/10.1016/j.jisp.2020.05.008 |
| El Backly, 2014 | No | Not related to the topic | No | Not related to the topic | DOI: https://doi.org/10.1016/j.jcms.2013.06.012 |
| El Bakly, 2020 | No | Not related to the topic | No | Not related to the topic | DOI: https://doi.org/10.1016/j.heliyon.2020.e05006 |
| El-Anwar, 2015 | No | Not related to the topic | No | Not related to the topic | DOI: https://doi.org/10.1016/j.anl.2015.02.016 |
| El-Sherbiny, 2022 | No | Not related to the topic | No | Not related to the topic | DOI: https://doi.org/10.1016/j.theriogenology.2022.09.029 |
| Elgazzar, 2008 | No | Not related to the topic | No | Not related to the topic | DOI: https://doi.org/10.1016/j.ijom.2008.05.010 |
| Elksniņš-Finogejevs, 2020 | No | Not related to the topic | No | Not related to the topic | DOI: 10.1186/s13018-020-01753-z |
| Engebretsen, 2012 | No | Not related to the topic | No | Not related to the topic | DOI: https://doi.org/10.1053/j.oto.2011.10.006 |
| Engineering, 2023 | No | Not related to the topic | No | Not related to the topic | DOI: 10.1155/2023/9758367 |
| Eryılmaz, 2016 | No | Not related to the topic | No | Not related to the topic | DOI: https://doi.org/10.1016/j.anl.2015.06.012 |
| Escamilla Cardeñosa, 2017 | No | Not related to the topic | No | Not related to the topic | DOI: https://doi.org/10.1016/j.jtv.2016.11.003 |
| Everts, 2023 | No | Not related to the topic | No | Not related to the topic | DOI: 10.3390/biomedicines11071922 |
| Farjah, 2020 | No | Not related to the topic | No | Not related to the topic | DOI: https://doi.org/10.1016/j.burns.2020.05.019 |
| Farkash, 2019 | No | Not related to the topic | No | Not related to the topic | DOI: https://doi.org/10.1016/j.jse.2018.09.007 |
| Farzadiniya, 2024 | No | Not related to the topic | No | Not related to the topic | DOI: https://doi.org/10.1016/j.jtv.2024.05.001 |
| Fei, 2021 | No | Not related to the topic | No | Not related to the topic | DOI: https://doi.org/10.1016/j.otsr.2021.103007 |
| Fennis, 2004 | No | Not related to the topic | No | Not related to the topic | DOI: https://doi.org/10.1054/ijom.2003.0452 |
| Galasso, 2008 | No | Not related to the topic | No | Not related to the topic | DOI: 10.1007/s10195-008-0021-7 |
| Gandhi, 2006 | No | Not related to the topic | No | Not related to the topic | DOI: https://doi.org/10.1016/j.bone.2005.10.019 |
| Gao, 2020 | No | Not related to the topic | No | Not related to the topic | DOI: https://doi.org/10.1016/j.burns.2019.07.041 |
| Garbis, 2011 | No | Not related to the topic | No | Not related to the topic | DOI: https://doi.org/10.1053/j.otsm.2011.03.002 |
| Garcia, 2017 | No | Not related to the topic | No | Not related to the topic | DOI: https://doi.org/10.1016/j.bjpt.2017.06.007 |
| García-Martínez, 2012 | No | Not related to the topic | No | Not related to the topic | DOI: https://doi.org/10.1016/j.joms.2011.06.199 |
| Garcia-Orue, 2022 | No | Not related to the topic | No | Not related to the topic | DOI: https://doi.org/10.1016/j.msec.2022.112695 |
| Hamilton, 2015 | No | Not related to the topic | No | Not related to the topic | DOI: 10.1136/bjsports-2012-091916 |
| Hamman, 2014 | No | Not related to the topic | No | Not related to the topic | DOI: https://doi.org/10.1016/j.amjcard.2013.12.046 |
| Han, 2013 | No | Not related to the topic | No | Not related to the topic | DOI: 10.7694/jldxyxb20130215 |
| Hancı, 2015 | No | Not related to the topic | No | Not related to the topic | DOI: https://doi.org/10.1016/j.jcms.2014.11.002 |
| Harna, 2022 | No | Not related to the topic | No | Not related to the topic | DOI: 10.3390/bioengineering10010021 |
| Hasley, 2023 | No | Not related to the topic | No | Not related to the topic | DOI: https://doi.org/10.1016/j.arrct.2023.100257 |
| Hassan, 2015 | No | Not related to the topic | No | Not related to the topic | DOI: https://doi.org/10.1016/j.ejr.2014.11.004 |
| Ibrahim, 2019 | No | Not related to the topic | No | Not related to the topic | DOI: https://doi.org/10.1016/j.ejr.2018.06.004 |
| Inbarajan, 2021 | No | Not related to the topic | No | Not related to the topic | DOI: 10.4103/jpbs.jpbs_74_21 |
| Johal, 2019 | No | Not related to the topic | No | Not related to the topic | DOI: 10.1177/1941738119834972 |
| Joshi, 2020 | No | Not related to the topic | No | Not related to the topic | DOI: 10.1200/JCO.2020.38.6_suppl.TPS380 |
| Jiritano, 2022 | No | Not related to the topic | No | Not related to the topic | DOI: 10.31083/j.fbe1402012 |
| Kanwat, 2017 | No | Not related to the topic | No | Not related to the topic | DOI: 10.11138/mltj/2017.7.3.426 |
| Kao, 2022 | No | Not related to the topic | No | Not related to the topic | DOI: 10.1177/23259671221088820 |
| Kao, 2021 | No | Not related to the topic | No | Not related to the topic | DOI: https://doi.org/10.1016/j.burns.2020.11.005 |
| Kapi, 2015 | No | Not related to the topic | No | Not related to the topic | DOI: 10.1097/sap.0000000000000160 |
| Karabaş, 2021 | No | Not related to the topic | No | Not related to the topic | DOI: https://doi.org/10.1016/j.transci.2020.103048 |
| Laidding, 2021 | No | Not related to the topic | No | Not related to the topic | DOI: https://doi.org/10.1016/j.mcpsp.2021.100239 |
| Landesberg, 2005 | No | Not related to the topic | No | Not related to the topic | DOI: https://doi.org/10.1016/j.joms.2004.12.007 |
| Lee, 2021 | No | Not related to the topic | No | Not related to the topic | DOI: https://doi.org/10.1016/j.ijpharm.2021.120242 |
| Leisi, 2023 | No | Not related to the topic | No | Not related to the topic | DOI: https://doi.org/10.1016/j.bjps.2022.11.059 |
| Mahendiran, 2022 | No | Not related to the topic | No | Not related to the topic | DOI: https://doi.org/10.1016/j.ijbiomac.2022.07.052 |
| Mahmoud, 2023 | No | Not related to the topic | No | Not related to the topic | DOI: https://doi.org/10.1016/j.archoralbio.2023.105674 |
| Maia, 2009 | No | Not related to the topic | No | Not related to the topic | DOI: https://doi.org/10.1016/j.jevs.2009.07.001 |
| Nakajima, 2024 | No | Not related to the topic | No | Not related to the topic | DOI: https://doi.org/10.1016/j.reth.2024.03.021 |
| Patel, 2022 | No | Not related to the topic | No | Not related to the topic | DOI: 10.1007/s43465-022-00737-x |
| Paterson, 2018 | No | Not related to the topic | No | Not related to the topic | DOI: 10.1186/s12891-018-2205-5 |
| Pundkar, 2024 | No | Not related to the topic | No | Not related to the topic | DOI: 10.7759/cureus.52229 |
| Qu, 2021 | No | Not related to the topic | No | Not related to the topic | DOI: https://doi.org/10.1016/j.mayocp.2021.01.030 |
| Reddy, 2023 | No | Not related to the topic | No | Not related to the topic | DOI: 10.1177/23259671221137923 |
| Redmond, 2015 | No | Not related to the topic | No | Not related to the topic | DOI: https://doi.org/10.1016/j.arthro.2014.08.034 |
| Shim, 2022 | No | Not related to the topic | No | Not related to the topic | DOI: https://doi.org/10.1016/j.jse.2021.10.036 |
| Smith, 2023 | No | Not related to the topic | No | Not related to the topic | DOI: https://doi.org/10.1016/j.inpm.2023.100237 |
| Stone, 2024 | No | Not related to the topic | No | Not related to the topic | DOI: 10.1177/03635465231185289 |
| Tang, 2022 | No | Not related to the topic | No | Not related to the topic | DOI: https://doi.org/10.1016/j.mtbio.2022.100498 |
| Tang, 2023 | No | Not related to the topic | No | Not related to the topic | DOI: https://doi.org/10.1016/j.ijbiomac.2023.125754 |
| Tey, 2022 | No | Not related to the topic | No | Not related to the topic | DOI: 10.1155/2022/3852898 |
| Tian, 2023 | No | Not related to the topic | No | Not related to the topic | DOI: https://doi.org/10.1016/j.mehy.2023.111097 |
| Zhao, 2020 | No | Not related to the topic | No | Not related to the topic | DOI: https://doi.org/10.1016/j.jot.2019.10.002 |
| Zhou, 2024 | No | Not related to the topic | No | Not related to the topic | DOI: https://doi.org/10.1016/j.freeradbiomed.2024.02.020 |
