# Supplementary material for: Efficacy of platelet-rich plasma in the treatment of erectile dysfunction: A meta-analysis of controlled and single-arm trials
Source: PLoS One. 2024 Nov 14;19(11):e0313074. doi: 10.1371/journal.pone.0313074 (PMC11563399; doi:10.1371/journal.pone.0313074)
Supplement: S2 Table — (DOCX) [file pone.0313074.s014.docx]

| **Research** | | **Included** | **Data extractor** | **Date of data extraction** | **Eligible** | **ΔIIEF** | | **MCID** | | **PSV** | | **EDV** | | **VAS** | | **Complication** | |
| --- | --- | --- | --- | --- | --- | --- | --- | --- | --- | --- | --- | --- | --- | --- | --- | --- | --- |
|  |  |  |  |  |  | **PRP** | **Non PRP** | **PRP** | **Non PRP** | **PRP** | **Non PRP** | **PRP** | **Non PRP** | **PRP** | **Non PRP** | **PRP** | **Non PRP** |
| RCT | Barone 2022 | Yes | Shaokang Du | 2024.3.30 | Confirm | 5.20±1.36 | 4.12±2.67 | - | - | - | - | - | - | - | - | - | - |
|  | Boyuk, 2022 | Yes | Shaokang Du | 2024.4.2 | Confirm | 5.20±1.46 | 4.06±0.30 | - | - | - | - | - | - | - | - | 2 | - |
|  | Epifanova, 2024 | Yes | Fuyu Guo | 2024.5.1 | Confirm | 7.00±4.53 | 5.66±6.55 | - | - | 28.78±8.75 | 24.92±6.57 | - | - | - | - | - | - |
|  | Geyik, 2021 | Yes | Fuyu Guo | 2024.4.5 | Confirm | 8.48±3.09 | 7.47±4.54 | 66/91 | 45/93 | - | - | - | - | - | - | - | - |
|  | Khalef, 2023 | Yes | Fuyu Guo | 2024.3.30 | Confirm | - | - | 35/42 | 5/ 17 | - | - | - | - | - | - | - | - |
|  | Ledesma, 2023 | Yes | Shaokang Du | 2024.4.2 | Confirm | 3.56±4.13 | 3.04±3.36 | 14/28 | 15/33 | 48.76±10.39 | 44.70±9.92 | 1.60±2.19 | 1.20±1.24 | - | - | - | - |
|  | Masterson, 2023 | Yes | Fuyu Guo | 2024.3.30 | Confirm | 4.40±7.60 | 2.90±5.60 | 12/20 | 10/24 | 49.30±11.10 | 45.10±15.10 | - | - | 4.10±2.30 | 4.00±2.50 | 1 | 1 |
|  | Poulios, 2021 | Yes | Shaokang Du | 2024.3.29 | Confirm | 3.30±4.00 | -0.20±3.80 | 20/29 | 7/26 | - | - | - | - | 2.20±0.60 | 2.60±0.40 | - | - |
|  | Ruffo, 2019 | Yes | Fuyu Guo | 2024.5.1 | Confirm | 7.97±4.77 | 4.44±4.58 | - | - | 49.60 ± 8.80 | 34.20 ± 9.70 | - | - | - | - | - | - |
|  | Ruffo, 2020 | Yes | Shaokang Du | 2024.4.2 | Confirm | 7.30±3.25 | 3.30±3.00 | - | - | 47.10±7.80 | 36.10±10.60 | - | - | - | - | - | - |
|  | Shaher, 2023 | Yes | Fuyu Guo | 2024.3.30 | Confirm | 2.90±5.09 | 0.00±3.82 | 35/50 | 8/50 | 32.72±3.92 | 19.72±5.50 | 1.71±1.53 | 6.00±1.53 | 1.52±1.20 | 1.54±1.30 | - | - |
| **Research** | | **Included** | **Data extractor** | **Date of data extraction** | **Eligible** | **Pre IVL** | **Post IVL** | **Pre IVL** | **Post IVL** | **Pre IVL** | **Post IVL** | **Pre IVL** | **Post IVL** | **Pre IVL** | **Post IVL** | **Pre IVL** | **Post IVL** |
| Single-arm trials | Achraf, 2023 | Yes | Shaokang Du | 2024.3.28 | Confirm | 6.30±2.10 | 7.60 ±2.50 | - | - | - | - | - | - | - | - | - | - |
|  |  |  |  |  |  | 5.10±2.50 | 6.80±3.20 | - | - | - | - | - | - | - | - | - | - |
|  | Banno, 2017 | Yes | Fuyu Guo | 2024.3.27 | Confirm | 15.78±2.68 | 19.51±5.35 | - | - | - | - | - | - | - | - | - | - |
|  | Epifanova, 2019 | Yes | Fuyu Guo | 2024.4.1 | Confirm | 12.40±2.91 | 18.60±2.59 | - | - | 29.87±11.15 | 39.69±10.09 | - | - | - | - | - | - |
|  | Epifanova, 2020 | Yes | Shaokang Du | 2024.3.29 | Confirm | 13.13±4.24 | 19.31±2.54 | - | - | 28.78±9.75 | 37.92±10.40 | - | - | - | - | - | - |
|  | Francomano, 2023 | Yes | Fuyu Guo | 2024.3.30 | Confirm | 11.95±2.54 | 18.60±3.21 | - | - | 31.98±5.92 | 41.86±6.33 | - | - | - | - | - | - |
|  | Schirmann, 2022 | Yes | Shaokang Du | 2024.4.2 | Confirm | 10.67±8.50 | 15.83±12.22 | - | - | - | - | - | - | - | - | - | - |
|  | Schirmann, 2022 | Yes | Shaokang Du | 2024.4.2 | Confirm | 11.80±5.51 | 15.15±6.44 | - | - | - | - | - | - | - | - | - | - |
|  | Taş, 2021 | Yes | Fuyu Guo | 2024.3.29 | Confirm | 16.22±11.27 | 17.15±10.88 | - | - | - | - | - | - | - | - | - | - |
|  | Wong, 2021 | Yes | Fuyu Guo | 2024.4.3 | Confirm | 12.03±5.10 | 16.59±5.50 | - | - | - | - | - | - | - | - | - | - |
|  | Zaghloul, 2021 | Yes | Shaokang Du | 2024.4.1 | Confirm | 7.71±2.74 | 13.21±6.77 | - | - | 49.17±15.87 | 50.69±17.45 | - | - | - | - | - | - |
