# Supplementary material for: Efficacy of platelet-rich plasma in the treatment of erectile dysfunction: A meta-analysis of controlled and single-arm trials
Source: PLoS One. 2024 Nov 14;19(11):e0313074. doi: 10.1371/journal.pone.0313074 (PMC11563399; doi:10.1371/journal.pone.0313074)
Supplement: S3 Table — (DOCX) [file pone.0313074.s015.docx]

| **Research** | **Random sequence generation (selection bias)** | **Allocation concealment (selection bias)** | **Blinding of participants and personnel (performance bias)** | **Blinding of outcome assessment (detection bias)** | **Incomplete outcome data (attrition bias)** | **Selective reporting (reporting bias)** | **Other bias** |
| --- | --- | --- | --- | --- | --- | --- | --- |
| Barone 2022 | Unclear Risk | Unclear Risk | Unclear Risk | Unclear Risk | Low Risk | Low Risk | Unclear Risk |
| Boyuk, 2022 | Unclear Risk | Unclear Risk | Unclear Risk | Unclear Risk | Low Risk | Low Risk | Unclear Risk |
| Epifanova, 2024 | Unclear Risk | Unclear Risk | Unclear Risk | Unclear Risk | Low Risk | Low Risk | Unclear Risk |
| Geyik, 2021 | Unclear Risk | Unclear Risk | High Risk | High Risk | Low Risk | Low Risk | Low Risk |
| Khalef, 2023 | Unclear Risk | Unclear Risk | Unclear Risk | Unclear Risk | Low Risk | Low Risk | Unclear Risk |
| Ledesma, 2023 | Low Risk | Unclear Risk | Unclear Risk | Unclear Risk | Low Risk | Low Risk | Unclear Risk |
| Masterson, 2023 | Low Risk | Low Risk | Low Risk | Low Risk | Low Risk | Low Risk | Low Risk |
| Poulios, 2021 | Low Risk | Low Risk | Low Risk | Low Risk | Low Risk | Low Risk | Low Risk |
| Ruffo, 2019 | Low Risk | Unclear Risk | Unclear Risk | Unclear Risk | Unclear Risk | Low Risk | Unclear Risk |
| Ruffo, 2020 | Unclear Risk | Unclear Risk | Unclear Risk | Unclear Risk | Low Risk | Low Risk | Unclear Risk |
| Shaher, 2023 | Low Risk | Low Risk | Low Risk | Low Risk | Low Risk | Low Risk | Low Risk |
